# Effect of use of NSAIDs or steroids during the acute phase of pain on the incidence of chronic pain: a systematic review and meta-analysis of randomised trials

**DOI:** 10.1007/s10787-023-01405-8

**Published:** 2023-12-28

**Authors:** Luyao Huo, Gang Liu, Bowen Deng, Lin Xu, Yanjun Mo, Shengyuan Jiang, Jingwei Tao, Huizhong Bai, Li Wang, Xiaoxiao Yang, Jizhou Yang, Xiaohong Mu

**Affiliations:** 1 Department of Orthopedics, Dongzhimen Hospital, Beijing University of Chinese Medicine, Beijing, China; 2 Division of Intelligent and Biomechanical System, State Key Laboratory of Tribology, Department of Mechanical Engineering, Tsinghua University, Haidian, Beijing, China; 3 Beijing An Yuan Quan Lv Medical Research Institute, Beijing, China; 4 Jinan Vocational College of Nursing, Jinan, China

**Keywords:** Anti-inflammatory drugs, Neuropathic pain, Postoperative pain, Chronic pain, Meta-analysis

## Abstract

**Background:**

This study is the first to summarize the evidence on how the use of anti-inflammatory drugs during acute pain has an impact on the development of chronic pain.

**Methods:**

Randomized controlled trials retrieved from nine databases included anti-inflammatory drugs (NSAIDs or steroids) versus non-anti-inflammatory drugs in patients with acute pain and reported the incidence of chronic pain. No specified date, age, sex, or language restrictions. Subgroup analyses were performed according to pain classification, follow-up time, and medication. The GRADE method was used to evaluate quality of evidence.

**Results:**

A total of 29 trials (5220 patients) were included. Steroids or NSAIDs did not reduce the incidence of chronic nociceptive pain. Steroid use in acute phase significantly reduced the incidence of chronic neuropathic pain. In subgroup analysis, benefits were observed for methylprednisolone and dexamethasone, with some adverse effects. Steroids or NSAIDs were statistically significant in reducing pain intensity over 1 year, but the effect size was too small, and whether the long-term effect is clinically relevant needs to be further studied.

**Conclusion:**

Quality of the evidence was low to moderate. No drug can be recommended to prevent chronic nociceptive pain. Injections of steroids (methylprednisolone or dexamethasone) during the acute phase reduce the incidence of chronic neuropathic pain, but most included studies also used local anesthetics. The results are indirect and need to be interpreted with caution. The pooled data effect sizes for pain intensity were small, so the clinical relevance was unclear.

*Study registration* PROSPERO (CRD42022367030).

**Supplementary Information:**

The online version contains supplementary material available at 10.1007/s10787-023-01405-8.

## Introduction

Once the pain lasts too long and far beyond the time for the body itself to recover, it becomes a burden (Clauw et al. 2019). The impact of even very low levels of pain on social and physical functioning is significant in terms of patients' attitudes towards their own health (Voscopoulos and Lema 2010). Chronic pain affects 8.3–13% of the population in the UK (Fayaz et al. 2016; Elliott et al. 2002). In the USA, about one in three people suffer from chronic pain, more than the number of people with cancer, diabetes and heart disease combined. It costs $635 billion a year (Gaskin and Richard 2012). The Global Burden of Disease Study 2016 again pointed out that pain and pain-related conditions are the leading causes of the global burden of disability and disease (GBD 2016 Disease and Injury Incidence and Prevalence Collaborators, 2017).

Chronic pain is defined as "pain which has persisted beyond normal tissue healing time (3 months)" according to the International Association for the Study of Pain (IASP) (Pain terms 1979; Treede et al. 2019). A study by Marc Parisien (Parisien et al. 2022) found that the use of anti-inflammatory drugs (steroids or NSAIDs) hindered pain recovery during acute pain phase. This study's conclusions are groundbreaking. The results should be replicated before major changes to current clinical practice can be made.

Chronic pain is a priority in post-acute and long-term care (Drake et al. 2019). Epidemiological studies have explored the risk factors for the development of chronic pain (Mills et al. 2019). While, few interventions to prevent chronic pain have been identified (Gewandter et al. 2015). No previous study has evaluated the effect of the use of anti-inflammatory drugs during the acute phase on the incidence of chronic pain. To improve the knowledge based on existing evidence, explore how the use of anti-inflammatory drugs during acute pain affects the development of chronic pain, we designed and conducted this study.

## Methods

### Study eligibility

Trials were eligible for inclusion if they: (1) Public published or registered with ClinicalTrials.gov, full data available, randomized, controlled, parallel designed trials with a follow-up of at least 3 months [pain lasting more than 3 months was defined as chronic pain (Treede et al. 2019)]. (2) The types of pain studied were nociceptive pain (including pain in the bones, muscles and skin, such as acute post-operative pain (APSP), etc. (Cohen et al. 2021)) or neuropathic pain [such as herpes zoster, acute lumbar radiculopathy, etc. (Finnerup et al. 2016)], with a duration of no more than 3 months before the first visit. (3) Anti-inflammatory drugs (NSAIDs or steroids) versus any non-anti-inflammatory control. (4) Outcome measure must include the incidence of chronic pain or pain intensity. Studies were excluded if they: (1) were animal or cell experiments, (2) were nonpharmacologic or follow-up time was less than 3 months. (3) Were cancer pain, bacterial infections, visceral pain, muscle cramps and nociplastic pain. (4) The type of pain could not be accurately resolved. This study is registered with PROSPERO (ID CRD42022367030). We anticipated that there would be great heterogeneity in the retrieved literature in terms of pain types and interventions, so we adjusted the original protocol before analysis including screening studies and subgroup analyses.

### Search strategy

This study followed the PRISMA Statement. One investigator (HLY) searched 9 databases: PubMed, Web of Science, Embase, the Cochrane Library, ClinicalTrials.gov, CBMdisc, CNKI, Wanfang Database and VIP. No specified date, age, sex, or language restrictions (search strategy is shown in supplementary Table 1). We also searched cited references of relevant trial reports and reviews for potentially eligible studies.

### Selection of studies

After deleting the duplicate literatures through Noteexpress (v3.9.0.9588) software, two reviewers (HLY, LG) independently screened all the study by reading the title and abstract. All studies deemed eligible after reading the title and abstract were reviewed by two pairs of reviewers (HLY, BHZ, WL, TJW) to determine whether they met the inclusion criteria. Disagreements were resolved by consensus or by consulting an adjudicator (MXH or YJZ).

### Data extraction

Two paired reviewers (MYJ, JSY; HLY, LG) independently extracted this information from each study. Data extraction tables were designed to extract study characteristics and outcome information as recommended in the Cochrane Handbook (Higgins et al. 2022): year of publication, authors, journal, geographic location, funding source, design, participants, cohort size, aims and intervention, follow-up time point and outcomes. If the data of the study were represented by figures, the data were extracted by two rreviewers (HLY, LG) using data extraction software (EngaugeDigitizer 11.3). For dates not reported, the two reviewers (HLY, LG) calculated from the available data according to the conversion formula suggested in Cochrane Handbook.

### Outcomes of interest

The primary outcome is the number of participants with persistent pain (three months or greater). The secondary outcomes was adverse events.

### Risk of bias assessment

The risk of bias in the included studies was assessed by two researchers(MYJ, YXX) using the Cochrane Collaboration Risk of Bias Tool (Sterne et al. 2019), for risk assessment of bias in RCT. The evaluator should make a low bias risk, high bias risk and unclear judgment for each project. Disagreements were resolved by consensus or by consulting an adjudicator (XL).

### Data analysis

RevMan 5.3 software was used for meta-analysis of the data. Comparisons were 2-tailed using a *P* < 0.05 threshold.The effect size was pooled across trials using a random (I^2^ < 50%) or fixed (I^2^ > 50%) effects model and heterogeneity was expressed using the I^2^ statistic. For continuous data, standardized mean difference (SMD) or mean difference (MD) and 95% CI were used as the effect analysis statistic. For dichotomous data, we calculated relative risks (RR) and 95% CI on the basis of the frequency of events in each treatment group. The results of data analysis are presented using forest plots. Sensitivity analysis was performed by removing each study individually to assess the consistency and quality of the results.

### Subgroup analyses

Nociceptive and neuropathic pain were divided into two subgroups for analysis. Then we divided the above two subgroups into 1–2 months, 3 months, 6 months and 1–2 years according to the different follow-up time. Finally, steroids and NSAIDs were analyzed separately. Other confounding factors affecting the analysis such as causes of pain and interventions will also be included in different subgroups for analysis.

### Quality of evidence

We applied the GRADE approach to evaluate the overall quality of the evidence (Guyatt et al. 2008, b). Two independent reviewers (MXH, XL) assessed quality of the evidence from all studies and categorized it into four levels: high, moderate, low, and very low. Disagreements were resolved by consensus.

## Result

### Study selection and characteristics

**Fig. 1:**
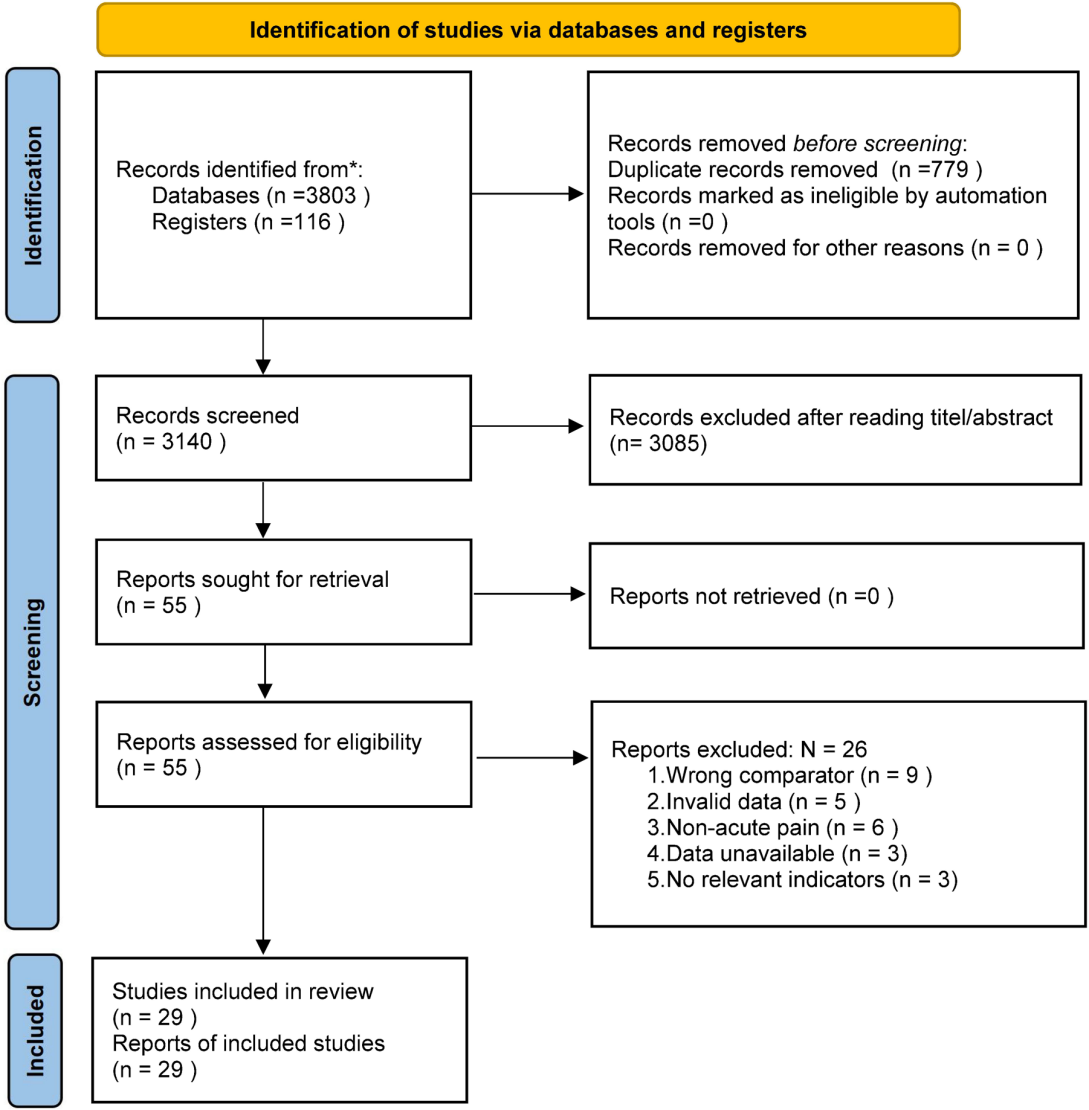
Flow diagram of study selection

A total of 3803 articles were retrieved in nine databases, and 116 articles were found through other sources such as references. After removing 779 duplicate articles, the remaining 3140 articles were screened. Finally, 29 eligible articles were included (Fig. 1). Table 1 summarizes the characteristics of the studies included in the evidence synthesis. The duration of pain was less than 3 months in all participants before the first visit.

**Table 1:** Characteristics of included studies

References	Country	Participants				Intervention			Follow-up time points	Outcome related to this study	Adverse events
		Duration of pain	Sampl size (n)	Age (years)	Durg	Single dose/Administration	Duration of intervention	Control			
Turan et al. (2015)	USA China Canada Australia	APSP, 0 day	1043	71.25 ± 11.55	Methylprednisolone	250 mg/iv	Single	Placebo	1 m, 6 m	Persistent incisional pain at 30 d (%) and 6 mo (%)	Not report
Bugada et al. (2015)	Italy	APSP, 0 day	194	57 ± 14.96	Ketorolac	30 mg/iv	4d	Tramadol 100 mg given comparably	1 m, 3 m	Proportion of patients with the long-term pain	1 case constipation, 1 case of bleeding (anal rhagade bleeding) and 2 cases of gastric symptoms
Comez et al. (2015)	Turkey	APSP, 0 day	60	47.12 ± 19	Dexketoprofen	50 mg/iv	15 min before surgery or 12 h later	levobupivacaine 0.125% 10 ml + Fentanyl citrate 50–100 mcg/h	1 m, 3 m, 6 m	VAS	Not report
Sun et al. (2013)	China	APSP, 0 day	60	45.45 ± 11	Flurbiprofen axetil	50 mg/iv	15 min before surgery and 6 h later	Placebo	2 m, 4 m, 6 m, 12 m	NRS Incidence of pain %	None
Ling et al. (2016)	China	APSP, 0 day	83	56.51 ± 11.23	Parecoxib	40 mg/iv	30 min before surgery and every 12 h for 60 h	Placebo	3 m, 12 m	Incision related pain (%)	5 Hypotension, 3 Dizziness, 6 Nausea and vomitting
van Helmond et al. (2016)	Netherlands	APSP, 0 day	94	52.96 ± 10.17	Parecoxib + Celecoxib	40 mg iv./200 mg po	iv.:30 min before surgery and 6 h later po.:every morning for 5 days	Placebo	1 m, 3 m, 6 m, 12 m	VAS rest (Data were extracted on pictures and the VAS move interval was too close to extract)	Not report
Fransen et al. (2006)	Australia New Zealand	APSP, 0 day	998	66.5 ± 11.52	Ibuprofen	400 mg/po	3 times a day for 14 days	Placebo	Between 6 and 12 m	Pain intensity (range of 0–10) At least daily analgesics for hip pain %	Bleeding
Romundstad et al. (2006)	Norway	APSP, 0 day	219	28.68 ± 6.77	Methylprednisolone/Parecoxib split into two studies	125 mg iv./40 mg iv	Single	Placebo	1.5 m, 12 m	Proportion of patients with the long-term pain	Not report
Benoldi et al. (1991)	Italy	< 72 h	36	66.45	Prednisone	35 mg/po	10 days and then gradually reduced to zero over 3 weeks	Standard treatment	6 m	Proportion of patients with the long-term pain	None
van Wijck et al. (2006)	Netherlands	< 7 days	598	66.5 ± 4.25	Methylprednisolone	80 mg/injected epidurally	Single	Standard treatment	1 m, 3 m, 6 m	Presence and severity of pain at each time points	6 dizziness, 3 flushes, 8 headache, 15 backache
Hancock et al. (2007)	Australia	< 6 weeks	239	40.7 ± 15.6	Diclofenac	Not reported/PO	Twice a day, until the patient had recovered or for a maximum of 4 weeks	Spinal manipulative therapy two or three times per week to a maximum of 12 treatments over 4 weeks + or Placebo	1 m, 3 m	Number at risk	11 gastrointestinal disturbances, dizziness, and heart palpitations
Bogefeldt et al. (2008)	Sweden	< 3 months	160	41.33 ± 8.51	Steroid (Names were not reported)	Not reported/injection	1 to 4 times	Physical activities, Medical exercise therapy, sequential exercise, non-specific traction, Passive treatment modalities, muscle stretching and spinal manipulation in 10 weeks	3 m, 2y	Sick leave rate at 10 weeks and 2 years	Not report
Dojode (2012)	India	7–9 weeks	60	42.55 ± 1.16	Methylprednisolone	80 mg/local injection	Not reported	2 ml autologous blood + 1 ml 0.5% bupivacaine	1 m, 3 m, 6 m	VAS	None
Lin et al. (2010)	China	< 3 months	17	45.21 ± 9.36	Triamcinolone	40 mg/local injection	Not reported	Receiving injection with 1 ml 0.9% NaCl and 50 U botulinum toxin type A	1 m, 3 m	VAS	None
Makharita et al. (2012)	Egypt	< 2 weeks	61	60.11 ± 2.76	Dexamethasone	8 mg/stellate ganglion injections	2 times 1 week apart	Standard treatment + 8 ml saline twice daily stellate ganglion block	1 m, 3 m, 6 m	VAS Incidence of persistent herpetic pain	Drowsiness, 1 lower limbs edema
Shin et al. (2013)	Korea	< 4 weeks	58	38.31 ± 7.97	Diclofenac	75 mg/im	Not reported	Motion style acupuncture treatment	1 m, 6 m	NRS Low Back Pain NRS Leg Pain Split into two studies	Not report
Wang et al. (2014)	USA	APSP, 0 day	57	—	Ketorolac	2 mg/intrathecal	Single	Intrathecal 13.5 mg hyperbaric bupivacaine spinally plus 0.4 ml saline	2 m, 6 m	Proportion of non-zero pain	Not report
Spijker-Huiges et al. (2014)	Netherlands	< 4 weeks	63	43.7 ± 9.8	Triamcinolone	80 mg/injected epidurally	Not reported	Care as usual	1 m, 3 m, 6 m, 1y	NRS back pain NRS leg pain split into two studies	None
Haddad et al. (2019)	Tunis	APSP, 0 day	60	49.14 ± 11.48	Parecoxib	40 mg/iv	30 min before induction of anesthesia	Placebo	1y	Number of postoperative chronic pain patients	None
Saied et al. (2015)	Iran	0 day	82	—	Betamethasone	Not reported/local injection	Not reported	Closed reduction and percutaneous pin fixation + injection of water 2 cc into TFCC	3 m, 6 m	VAS Number of painless patients	Not report
Makharita et al. (2015)	Egypt	< 7 days	138	56.54 ± 3.41	Dexamethasone	8 mg/paravertebral injection	Single	Standard treatment + paravertebral injection of 10 mL saline	1 m, 3 m, 6 m	VAS	
Incidence of persistent herpetic pain	No serious adverse cardiovascular events, Drowsiness										
Goldberg et al. (2015)	USA	< 3 months	269	46.0 ± 12.1	Prednisone	60 mg, 40 mg, 20 mg/PO	15 days, each dose for 5 days	Standard treatment + placebo	1 m, 1y	NRS Proportion of participants achieving at least a 5 point improvement in the pain NRS scores	46 Insomnia, 33 Nervousness, 40 Increased appetite, 20 Indigestion, 32 Headache, 10 Joint pain, 35 Joint pain
Mardani-Kivi et al. (2015)	Iran	< 6 weeks	84	44.29 ± 8.54	Methylprednisolone	40 mg/local injection	Not reported	Extracorporeal shock wave therapy, energy level of 0.15 mJ/mm2, 2000 shock wave impulses were applied for 3 times at weekly intervals	1 m, 3 m	VAS	Not report
Cui et al. (2017) (A)	China	< 7 days	93	63.35 ± 3.74	Methylprednisolone	40 mg/Intracutaneous injection	Every 48 h for a week, total 4 injections	Standard treatment	1 m, 3 m, 6 m	VAS Incidence of postherpetic neuralgia	Bruising
Li et al. (2018)	China	APSP, 0 day	52	60.26 ± 9.33	Dexamethasone	10 mg/thoracic paravertebral block	Single	Placebo given comparably	3 m	VRS Incidence chronic pain	None
Cui et al. (2018) (B)	China	< 7 days	97	61.75 ± 7.27	Methylprednisolone	40 mg/Intracutaneous injection	Single	Standard treatment + Intra-cutaneous injection of 15 ml normal saline	1 m, 3 m, 6 m	VAS Incidence of postherpetic neuralgia	Transient burning pain
Ge et al. (2021)	China	APSP, 0 day	105	65.15 ± 6.52	Parecoxib	40 mg/iv	Once every 12 h until the sixth dose	4 ml normal saline given comparably	3 m	Proportion of patients with chronic post-surgical pain at 3 months	None
El-Sayed et al. (2021)	Egypt	< 3 months	40	20—60	Methylprednisolone	40 mg/Erector spinae plane block	Single. Interval was repeated every two weeks if VAS > 6	Standard treatment	1 m, 3 m	Incidence of postherpetic neuralgia	Not report
Ni et al. (2017)	China	< 7 days	100	64.85 ± 10.19	Triamcinolone	10 mg/Subcutaneous injection	Once per week for 3 weeks	Standard treatment	1 m, 3 m, 6 m	Incidence of postherpetic neuralgia	8 (16%) patients had self-limiting subcutaneous hemorrhage and 7 (14%) complained of pain at the injection point

APSP = Acute Postoperative pain; cc = cubic centimeter; h = hour; iv. = Intravenous injection; kg = kilogram; mg = milligram; ml = milliliter; m = month; NRS = numerical rating scale; n = number; po. = Oral administration (Peros); TFCC = triangular fibrocartilage complex; UK = United Kingdom; US = United States; VAS = visual analogue scale; y = year

### Risk of bias and grading of evidence assessment

Eighteen studies had a low risk of bias, 3 studies had an unclear risk of bias, and 8 studies had a high risk of bias (supplementary Fig. 1). Quality of the evidence was moderate overall (Table 2). The heterogeneity and imprecision of the combined effect size was the main reason for the downgrade.

**Table 2:** GRADE evidence profile

Time	Trials (n)	Patients (n)	Risk of bias	I^2^	Indirectness/imprecision	Likelihood of publication bias	Effect size in SMD/WMD (95% CI) or RR (95% CI)	Quality of evidence
Number of patients with persistent pain								
Nociceptive pain—steroids								
1–2 m	2	1160	No	0%	↓	No	0.99 [0.87, 1.12]	Moderate
3 m	3	294	No	37%	No	No	0.71 [0.53, 0.94]	High
6 m	2	1125	No	0%	↓	No	1.00 [0.80, 1.27]	Moderate
1–2 y	2	278	↓	17%	↓	No	0.65 [0.34, 1.25]	Low
Total	5	2857	No	13%	↓	No	0.94 [0.85, 1.04]	Moderate
Nociceptive pain—NSAIDs								
1–2 m	4	611	↓	0%	↓	No	0.92 [0.76, 1.11]	Low
3 m	5	679	↓	64%↓	↓	No	0.88 [0.55, 1.42]	Very Low
6 m	3	959	↓	67%↓	↓	No	0.51 [0.15, 1.76]	Very Low
1–2 y	4	304	↓	0%	No	No	0.38 [0.22, 0.65]	Moderate
Total	9	2553	↓	54%↓	↓	No	0.80 [0.64, 1.00]	Very Low
Neuropathic pain—steroids								
1–2 m	7	1323	No	84%↓	↓	No	0.50 [0.34, 0.75]	Low
3 m	7	1070	No	58%↓	↓	No	0.41 [0.24, 0.72]	Low
6 m	7	1051	No	43%	↓	No	0.43 [0.23, 0.81]	Moderate
1–2 y	1	234	No	–	–	No	–	–
Total	9	3444	No	72%↓	↓	No	0.47 [0.36, 0.62]	Low
Intensity of pain								
Nociceptive pain—steroids								
1–2 m	4	208	↓	69%↓	No	No	− 0.65 [− 1.18, − 0.11]	Low
3 m	6	342	↓	77%↓	↓	No	− 0.36 [− 0.83, 0.10]	Very Low
6 m	3	205	No	33%	No	No	− 0.38 [− 0.72, − 0.04]	High
1–2 y	1	63	No	–	–	–	–	–
Total	6	775	↓	67%↓	No	No	− 0.46 [− 0.72, − 0.19]	Low
Nociceptive pain—NSAIDs								
1–2 m	3	212	No	0%	No	No	− 0.38 [− 0.66, − 0.11]	High
3 m	3	214	↓	0%	No	No	− 0.52 [− 0.80, − 0.24]	Moderate
6 m	5	1120	No	79%↓	↓	No	− 0.28 [− 0.65, 0.10]	Low
1–2 y	1	94	No	–	–	–	–	–
Total	5	1546	No	75%↓	No	No	− 0.29 [− 0.54,− 0.04]	Moderate
Neuropathic pain—steroids								
1–2 m	6	719	No	52%↓	↓	No	− 0.99 [− 1.32, − 0.66]	Low
3 m	5	452	No	25%	↓	No	− 0.61 [− 0.91, − 0.31]	Moderate
6 m	5	452	No	0%	↓	No	− 0.40 [− 0.64, − 0.16]	Moderate
1–2 y	2	297	No	0%	↓	No	− 0.78 [− 1.48, − 0.07]	Moderate
Total	6	1920	No	38%	↓	No	− 0.61 [− 0.77, − 0.45]	Moderate
Neuropathic pain—NSAIDs								
Total	1	58	No	–	–	–	–	–

n = Number; –: data analysis could not be performed; ↓: the quality of evidence has dropped one level; m = month; y = year; SMD = standardized mean differences; WMD = weighted mean difference; RR = relative risk

### Nociceptive pain

Of the 20 studies (3788 participants) involving nociceptive pain, 1 study evaluated both steroids and NSAIDs (in different group) (Romundstad et al. 2006), 9 studies evaluated steroids (Turan et al. 2015; Romundstad et al. 2006; Bogefeldt et al. 2008; Dojode 2012; Lin et al. 2010; Spijker-Huiges et al. 2014; Saied et al. 2015; Mardani-Kivi et al. 2015; Li et al. 2018), and 12 studies evaluated NSAIDs (van Helmond et al. 2016; Sun et al. 2013; Romundstad et al. 2006; Ling et al. 2016; Fransen et al. 2006; Comez et al. 2015; Bugada et al. 2015; Hancock et al. 2007; Shin et al. 2013; Wang et al. 2014; Haddad et al. 2019; Ge et al. 2021).

#### The number of participants with persistent pain

##### Steroids

**Fig. 2:**
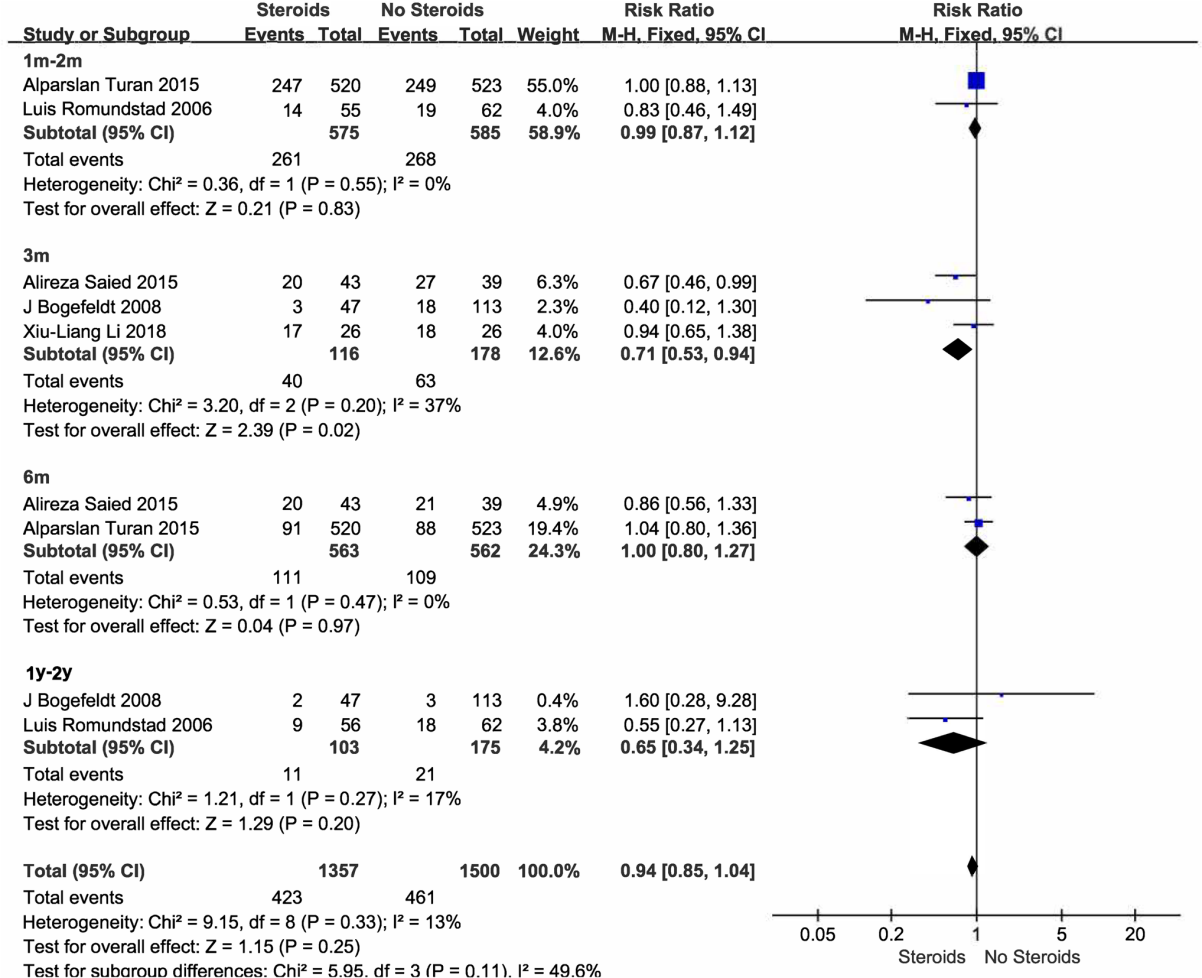
Steroids and incidence of chronic nociceptive pain

Of the 9 studies involving steroids, 5 studies (1556 participants) reported the incidence of chronic pain (Turan et al. 2015; Romundstad et al. 2006; Bogefeldt et al. 2008; Saied et al. 2015; Li et al. 2018). Moderate quality evidence supports that steroid use during the acute phase of pain does not reduce the incidence of chronic nociceptive pain compared with control (RR 0.94 [95% CI 0.85–1.04], *P* = 0.25, I^2^ = 13%; Fig. 2), with no reported adverse events. Subgroup analyses of the three studies (Turan et al. 2015; Romundstad et al. 2006; Li et al. 2018) involving postoperative pain (n = 1314) showed no difference in the effect of steroids on the incidence of chronic pain compared with the control group (RR 0.98 [95% CI 0.88–1.09]; supplementary Fig. 2). Two other small sample studies (Bogefeldt et al. 2008; Saied et al. 2015) of non-postoperative pain (n = 242) showed that steroids can reduce the incidence of chronic pain, but neither reported therapeutic doses (RR 0.72 [95% CI 0.54–0.96]; supplementary Fig. 3), results need to be interpreted with caution.

The steroids evaluated included 2 methylprednisolone (125 mg/250 mg iv., single dose) (Turan et al. 2015; Romundstad et al. 2006), 1 betamethasone (1 cc local injection, duration unknown) (Saied et al. 2015), 1 dexamethasone (10 mg thoracic paravertebral block, single dose) (Li et al. 2018), 1 no specific medications and dose were not reported (Bogefeldt et al. 2008). No serious drug-related complications in the five studies. Subgroup analysis of the long-term outcomes of methylprednisolone (n = 1262) showed that a single dose (125 mg/250 mg iv.) did not reduce the incidence of chronic nociceptive pain compared with placebo (RR 0.83 [95% CI 0.46–1.50], supplementary Fig. 4).

##### NSAIDs

**Fig. 3:**
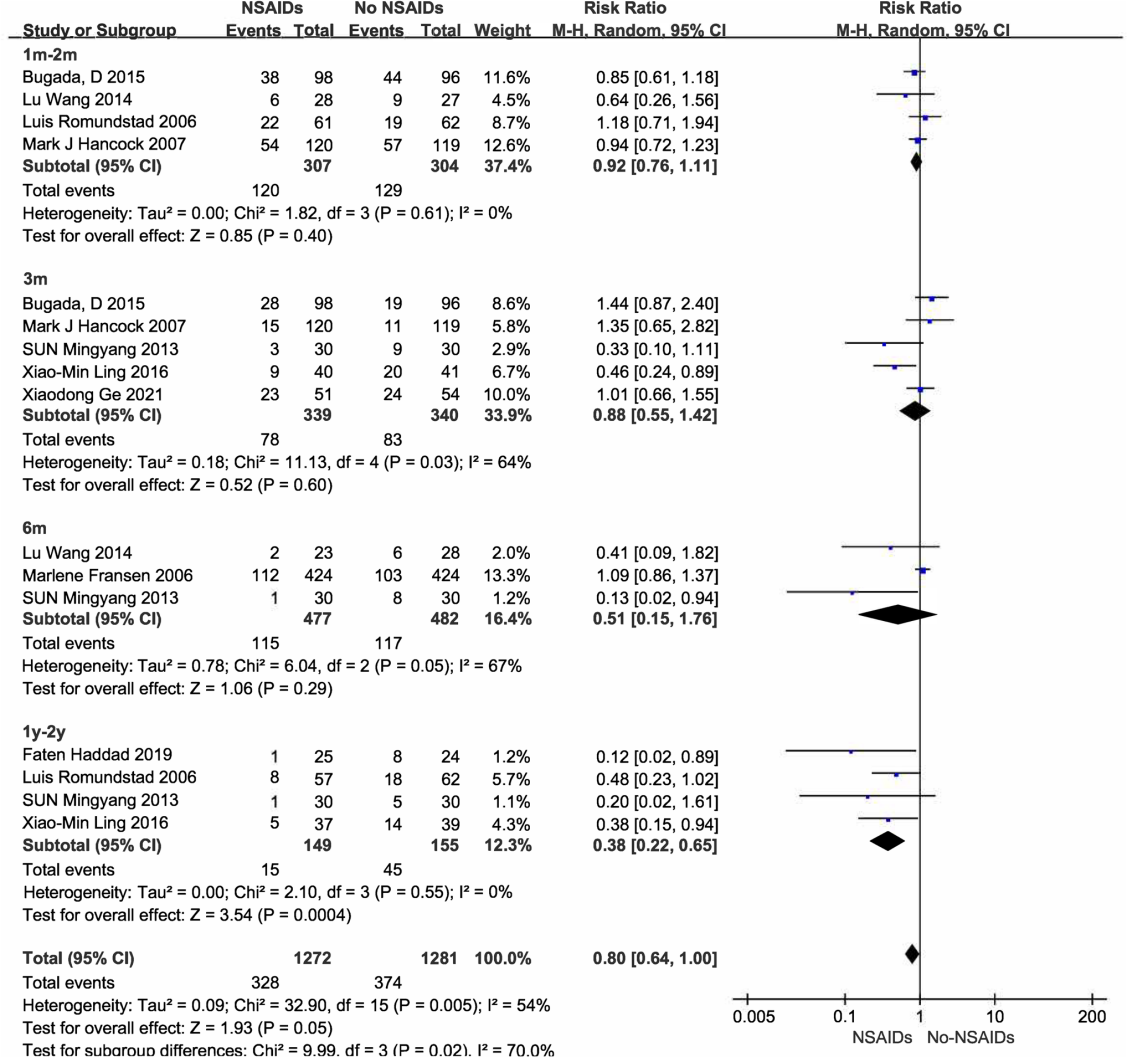
NSAIDs and incidence of chronic nociceptive pain

Of the 12 studies involving NSAIDs, 9 studies (2015 participants) reported the incidence of chronic pain (Sun et al. 2013; Romundstad et al. 2006; Ling et al. 2016; Fransen et al. 2006; Bugada et al. 2015; Hancock et al. 2007; Wang et al. 2014; Haddad et al. 2019; Ge et al. 2021). Very-low quality evidence supports that NSAIDs use during the acute phase of pain does not reduce the incidence of chronic nociceptive pain compared with control (RR 0.80 [95% CI 0.64–1.00], *P* = 0.05, I^2^ = 54%; Fig. 3). Subgroup analysis of 8 studies (n = 1776) involving postoperative pain showed a benefit of NSAIDs in reducing the incidence of chronic pain during the one-year follow-up period (RR 0.72 [95% CI 0.55–0.96]; supplementary Fig. 5), although this benefit was observed only at the one-year time point (n = 304, *P* for interaction = 0.04). The quality of evidence is very low and the results need to be interpreted with caution. Only one (Hancock et al. 2007) of the 9 studies involved non-postoperative pain and subgroup analysis was not possible.

Of the 4 studies followed for 1–2 years, 3 reported parecoxib (40 mg iv.) (Ling et al. 2016; Romundstad et al. 2006; Haddad et al. 2019). Two single dose studies had no adverse effects (Haddad et al. 2019; Romundstad et al. 2006) and one study in which five doses were administered within 60-h had hypotension, dizziness, nausea and vomiting but no significant difference in adverse events between the two groups (Ling et al. 2016). Another study reported flurbiprofen axetil (50 mg iv., 2 doses within 12 h) without adverse effects (Sun et al. 2013).

The NSAIDs reported in the 9 studies included 4 parecoxib (40 mg iv., single doses/6 doses within 12 h) (Ling et al. 2016; Haddad et al. 2019; Romundstad et al. 2006; Ge et al. 2021), 2 ketorolac (30 mg iv. tid./first 24 h, 10 mg iv. tid./3 days; 2 mg single intrathecal injection) (Wang et al. 2014; Bugada et al. 2015), 1 flurbiprofen axetil (50 mg iv., 2 doses within 12 h) (Sun et al. 2013), 1 ibuprofen (400 mg po., tid./14 days) (Fransen et al. 2006), and 1 diclofenac (dose unknown po., bid./28 days) (Hancock et al. 2007). Subgroup analysis showed that neither parecoxi (n = 354, RR 0.55 [95% CI 0.29–1.06], supplementary Fig. 6) nor ketorolac (n = 251, RR 0.94 [95% CI 0.29–3.07], supplementary Fig. 7) reduced the incidence of chronic nociceptive pain.

#### Intensity of pain

##### Steroids

**Fig. 4:**
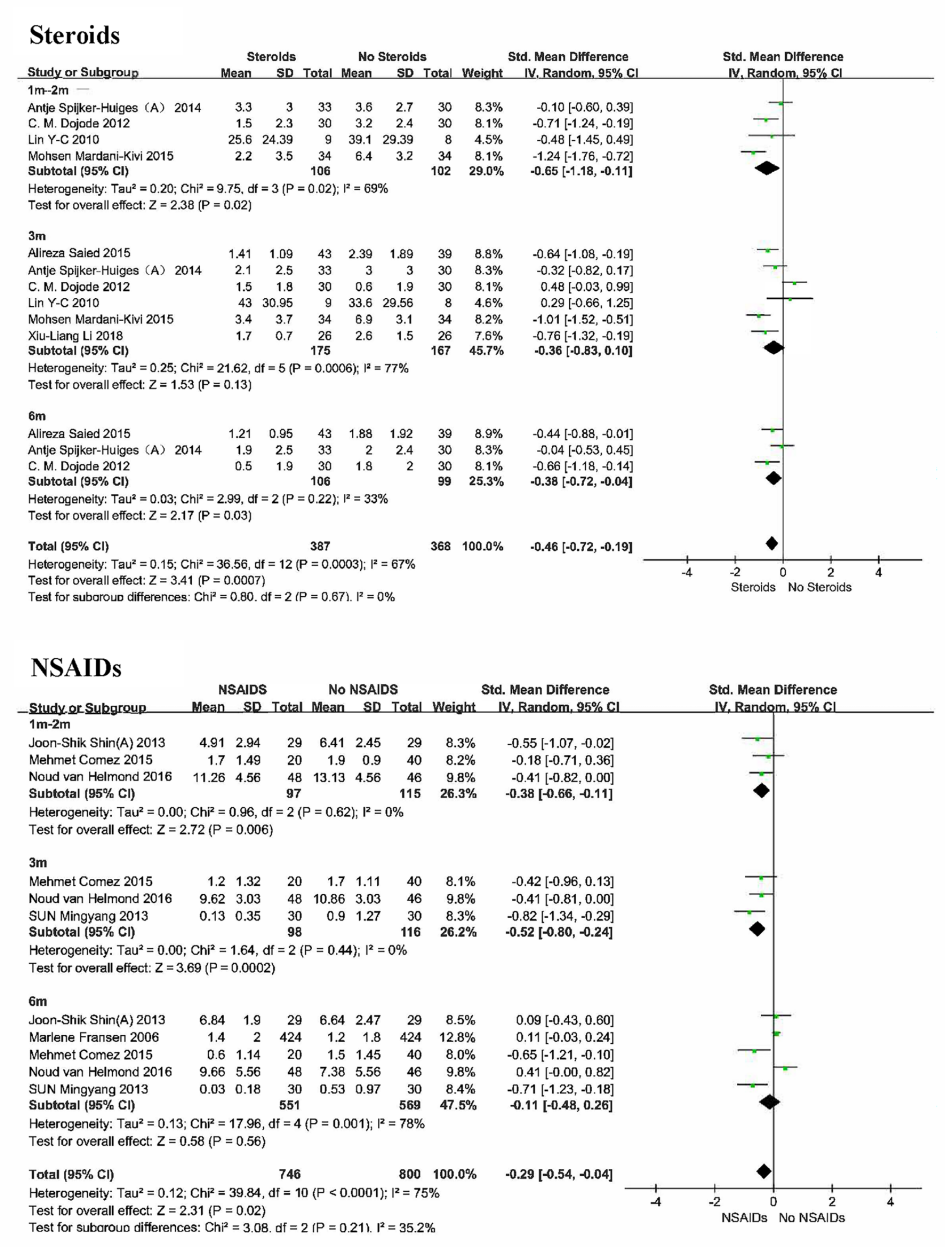
Steroids and NSAIDs and intensity of nociceptive pain

Of the 9 studies involving steroids, 6 studies (358 participants) reported intensity of pain (Dojode 2012; Lin et al. 2010; Spijker-Huiges et al. 2014; Saied et al. 2015; Mardani-Kivi et al. 2015; Li et al. 2018). Low quality evidence supported the use of steroids to reduce pain intensity in chronic nociceptive pain over the 6-month follow-up period (SMD − 0.46 [95% CI − 0.72 to − 0.19], *P* = 0.0007, I^2^ = 67%; Fig. 4), with no adverse effects observed. Subgroup analysis of 5 studies involving non-postoperative pain (n = 306) showed the same trend (SMD − 0.43 [95% CI − 0.71 to − 0.15]; supplementary Fig. 8). Only one (Li et al. 2018) of the 6 studies involved postoperative pain and subgroup analysis was not possible. There was considerable heterogeneity in the results. Sensitivity analysis showed that the heterogeneity was significantly reduced after removing the two studies (Dojode 2012; Mardani-Kivi et al. 2015). It may be caused by the significant difference in efficacy between the selected control groups (bupivacaine local injection, intermediate shock wave).

Reported drugs included 2 methylprednisolone (40 mg/80 mg local injection) (Dojode 2012; Mardani-Kivi et al. 2015), 2 triamcinolone (40 mg local injection/80 mg epidural injection) (Lin et al. 2010; Spijker-Huiges et al. 2014), 1 betamethasone (1 cc local injection) (Saied et al. 2015), and 1 dexamethasone (10 mg/thoracic paravertebral block, single dose) (Li et al. 2018). All six studies had small sample sizes, five of them did not report duration of medication intervention, and intervention methods in the control group varied considerably, so we did not perform subgroup analyses by drugs.

##### NSAIDs

Of the 12 studies involving NSAIDs, 5 studies (1270 participants) reported intensity of pain (van Helmond et al. 2016; Sun et al. 2013; Fransen et al. 2006; Comez et al. 2015; Shin et al. 2013). Moderate quality evidence supported the use of NSAIDs to reduce pain intensity in chronic nociceptive pain over the 6-month follow-up period (SMD − 0.29 [95% CI − 0.54 to − 0.04], *P* = 0.02, I^2^ = 75%; Fig. 4**)**, with bleeding as an adverse effect (ibuprofen 400 mg po., tid./14 days). Sensitivity analysis showed that heterogeneity was significantly reduced after excluding one studies (Fransen et al. 2006), from 75 to 61%. The other four studies were all administered by injection, and this study was administered by oral administration, which may be the cause of heterogeneity. Subgroup analysis of 4 studies involving postoperative pain (n = 1212) showed the same trend (SMD − 0.31 [95% CI − 0.59 to − 0.03]; supplementary Fig. 9). Only one (Shin et al. 2013) of the 5 studies involved non-postoperative pain and subgroup analysis was not possible.

Reported drugs included dexketoprofen (50 mg iv., 2 doses within 24 h) (Comez et al. 2015), flurbiprofen axetil (50 mg iv., 2 dose within 12 h) (Sun et al. 2013), parecoxib (40 mg iv., 2 dose within 12 h) + celecoxib (200 mg po., qd./5 days) (van Helmond et al. 2016), ibuprofen (400 mg po., tid./14 days) (Fransen et al. 2006) and diclofenac (75 mg im., duration unknow) (Shin et al. 2013). Subgroup analyses could not be performed to combine data for individual drugs.

### Neuropathic pain

Of the 11 studies (1553 participants) involving neuropathic pain, 10 studies evaluated steroids (Benoldi et al. 1991; van Wijck et al. 2006; Makharita et al. 2012, 2015; Spijker-Huiges et al. 2014; Goldberg et al. 2015; Cui et al. 2017, 2018; El-Sayed et al. 2021; Ni et al. 2017) and only 1 study evaluated NSAIDs (Shin et al. 2013).

#### The number of participants with persistent pain

**Fig. 5:**
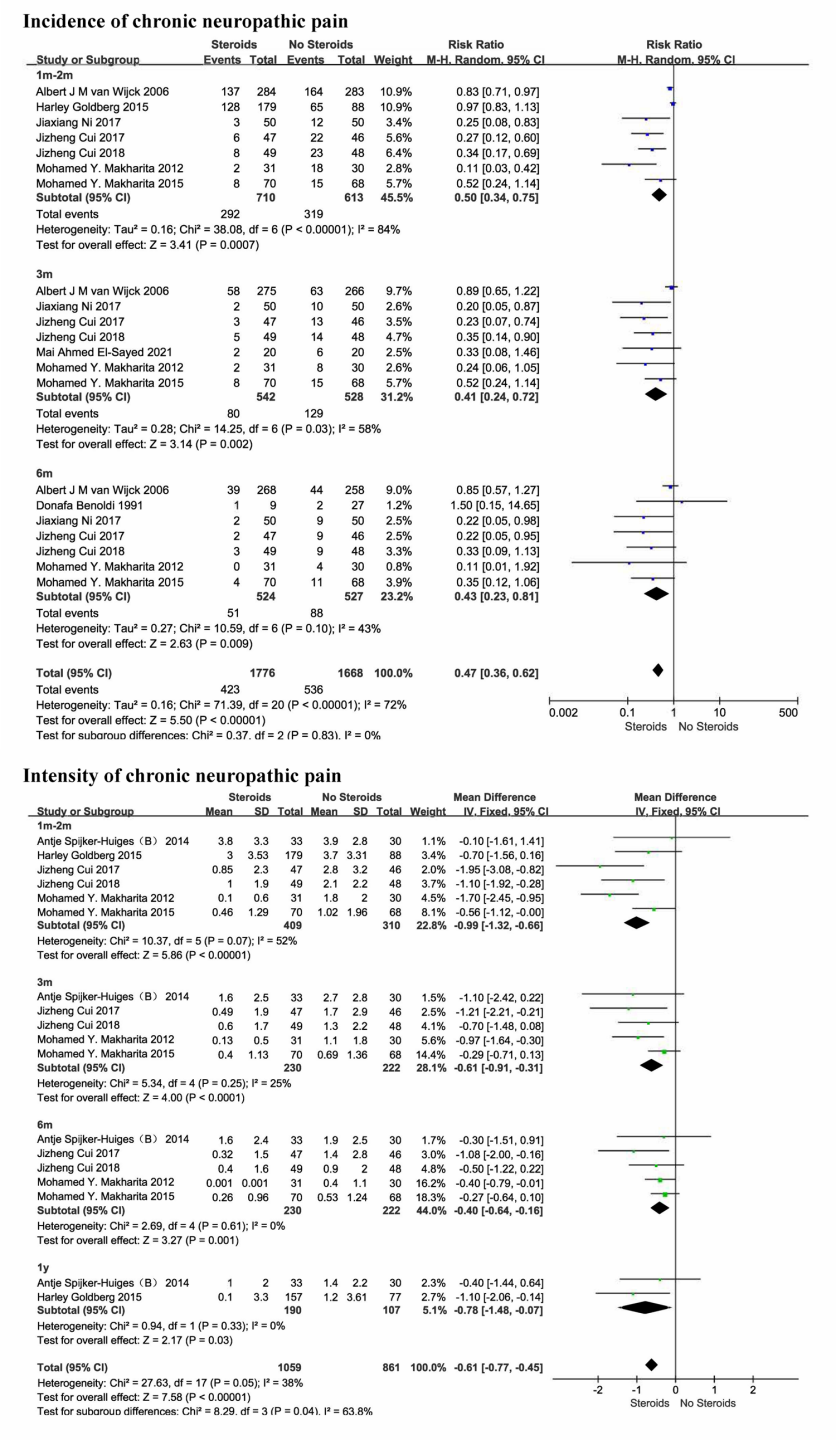
Incidence and intensity of chronic neuropathic pain

Of the 10 studies involving steroids, 9 studies (1432 participants) reported the incidence of chronic pain (Benoldi et al. 1991; van Wijck et al. 2006; Makharita et al. 2012, 2015; Goldberg et al. 2015; Cui et al. 2017, 2018; El-Sayed et al. 2021; Ni et al. 2017). Low quality evidence supports a significant reduction (53% lower) in the incidence of chronic neuropathic pain with the use of steroids in the acute phase compared with the control group during the 6-month follow-up period (RR 0.47 [95% CI 0.36–0.62], *P* < 0.0001, I^2^ = 72%; Fig. 5). Adverse reactions included insomnia, nervousness, increased appetite, dyspepsia, dizziness, headache, arthralgia, and arthralgia. The pooled results showed great heterogeneity, and sensitivity analysis showed that the heterogeneity was reduced to 65% when one study (Goldberg et al. 2015) was removed. This study looked at the effect of oral prednisone on acute radiculopathy, whereas the other studies were all acute herpes zoster and most were administered in combination with local anesthetic injections. Subgroup analysis of 8 studies (Benoldi et al. 1991; van Wijck et al. 2006; Makharita et al. 2012, 2015; Cui et al. 2017, 2018; El-Sayed et al. 2021; Ni et al. 2017) involving acute herpes zoster (n = 1163) showed that steroids significantly reduced the incidence of chronic pain (67% lower) over a 6-month follow-up period compared with usual care(RR 0.33 [95% CI 0.24–0.48]; supplementary Fig. 10).

The drugs reported included 4 methylprednisolone [40 mg intracutaneous injection, single (Cui et al. 2018) or 4 doses within 1 week (Cui et al. 2017)/erector spinae plane block, single (El-Sayed et al. 2021); 80 mg epidural injection, single dose (van Wijck et al. 2006)], 2 dexamethasone [8 mg paravertebral injection, single or 2 doses (Makharita et al. 2012, 2015)], prednisone [35 mg po., single dose for 10 days and reduced to 0 over next 21 days (Benoldi et al. 1991); 60 mg,40 mg, 20 mg, each dose for 5 days (Goldberg et al. 2015)] and 1 triamcinolone [10 mg subcutaneous injection, 3 doses within 3 weeks (Ni et al. 2017)]. With the exception of oral prednisone, steroid injections were combined with local anesthetics. This makes it difficult to evaluate the effect of a single steroid on the incidence of chronic neuropathic pain and downgrades the quality of evidence from moderate to low. Subgroup analyses (supplementary Figs. 11 and 12) showed that both methylprednisolone (n = 828, RR 0.52 [95% CI 0.38–0.73]) and dexamethasone (n = 199, RR 0.33 [95% CI 0.22–0.51]) combined with local anesthetic injection significantly reduced the incidence of chronic neuropathic pain and adverse effects of dizziness, headache, back pain, and somnolence were observed. However, adverse effects such as insomnia, nervus, increased appetite, dyspepsia, headache, and arthralgia were observed with multiple doses of oral prednisone, while no significant clinical benefit was observed (n = 305, RR 0.77 [95% CI 0.55–1.09], supplementary Fig. 13).

#### Intensity of pain

Of the 10 studies involving steroids, 6 studies (721 participants) reported intensity of pain (Makharita et al. 2012, 2015; Spijker-Huiges et al. 2014; Goldberg et al. 2015; Cui et al. 2017, 2018). Moderate quality evidence supports the use of steroids in the acute phase to reduce pain intensity, with statistically significant differences compared with controls (MD − 0.61 [95% CI − 0.77 to − 0.45], *P* < 0.0001, I^2^ = 38%; Fig. 5). Subgroup analysis of 4 studies (Makharita et al. 2012, 2015; Cui et al. 2017, 2018) involving acute herpes zoster (n = 1163; MD − 0.76 [95% CI − 1.04 to − 0.48]; supplementary Fig. 14) and 2 studies (Spijker-Huiges et al. 2014; Goldberg et al. 2015) involving acute radiculopathy (n = 332; MD − 0.67 [95% CI − 1.19 to − 0.16]; supplementary Fig. 15) showed the same trend. However, the pooled effect sizes were small, and whether the long-term benefits are clinically relevant is difficult to determine.

The drugs reported included 2 methylprednisolone (40 mg intracutaneous injection, single (Cui et al. 2017)/4 doses within 1 week (Cui et al. 2018)), 2 dexamethasone (8 mg paravertebral injection, single (Makharita et al. 2012)/2 doses (Makharita et al. 2015)), 1 prednisone (60 mg,40 mg, 20 mg, each dose for 5 days) (Goldberg et al. 2015) and 1 triamcinolone (80 mg epidural injection, unknown duration) (Spijker-Huiges et al. 2014). Subgroup analysis (supplementary Figs. 16 and 17) showed that both methylprednisolone (n = 190, MD − 0.97 [95% CI − 1.32 to − 0.61]) and dexamethasone (n = 199, MD − 0.61 [95% CI − 0.95 to − 0.26]) combined with local anesthetic injection could reduce the intensity of neuropathic pain during the 6-month follow-up period, and the adverse effects included sleepiness and lower limb edema. Individual studies of triamcinolone acetonide and prednisone could not be combined for data.

## Discussion

### Summary of evidence

Within 29 (n = 5220) randomized controlled trials, APSP was the main research model for nociceptive pain (n = 3788) and acute herpes zoster is for neuropathic pain (n = 1553). The synthesis of available evidence suggests that neither steroids (moderate quality) nor NSAIDs (very low quality) can be recommended to prevent chronic nociceptive pain during the acute phase of pain. The use of steroids in the acute phase significantly reduced the incidence of chronic neuropathic pain (53% lower, low quality). There are insufficient data to assess the association between NSAIDs and neuropathic pain. Both steroids (low quality) and NSAIDs (moderate quality) reduce pain intensity, but the combined effect sizes are small and there is substantial heterogeneity. Whether the long-term effect is clinically relevant needs to be further studied.

Subgroup analyses by cause of pain (postoperative or non-postoperative pain; acute herpes zoster or acute radiculopathy) showed the same trend as described above. Subgroup analyses showed that methylprednisolone (40 or 80 mg) and dexamethasone (8 mg) combined with local anesthesia injections significantly reduced the incidence and intensity of chronic neuropathic pain, with adverse effects including dizziness, headache, back pain, and somnolence. Administration methods include epidural injection, intradermal injection and paravertebral injection. Neither methylprednisolone (125 mg or 250 mg iv.), parecoxib (40mgiv.), nor ketorolac (30 mg iv./2 mg intradural injection) reduced the incidence of chronic nociceptive pain. Due to limitations in the number and design of the studies, we were unable to do separate subgroup analyses for all drugs.

### Limitations of previous studies

Previous studies have discussed the relationship between anti-inflammatory drugs and chronic nociceptive pain, mainly using postoperative pain as a research model. The study by Ian Gilron et al. (Carley et al. 2021), which reviewed the evidence for the use of drugs to prevent chronic nociceptive pain after surgery, showed that none of the drugs studied to date could be recommended. Compared with similar degrees of nociceptive pain, neuropathic pain might be associated with greater decrements in quality of life (Saavedra-Hernández et al. 2012; Spahr et al. 2017). However, there is little evidence summarizing the incidence of chronic neuropathic pain. Many epidemiological studies have explored the risk factors for the development of chronic pain, while some factors can identify the individuals who are at risk of developing chronic pain, few interventions to prevent chronic pain have been identified (Gewandter et al. 2015).

### Strengths and limitations of the study

There are some limitations to this meta-analysis. Twelve different drugs were used in the included studies, including five steroids and seven NSAIDs, and some were administered in different ways and for different durations. This leads to heterogeneity in partially integrated data, which is one of the main reasons for lowering the level of evidence. Studies of injectable administration in the treatment of neuropathic pain have all combined local anesthetics, making it difficult to assess the effect of steroids on the incidence of chronic neuropathic pain. We reduced the level of evidence due to indirectness. The incidence of chronic pain was not a primary outcome in some studies, which may be at risk for selective reporting. For pain intensity, the effect size in the pooled data was small and, although statistically significant, may not be clinically relevant. The small number of disease types included in the study is also a limitation of this study, with nociceptive pain mainly APSP and neuropathic pain mainly acute herpes zoster. Whether the conclusions can be generalized to pain caused by other diseases needs to be cautious. Some studies did not report the potential safety issues of the drug intervention, which was an impediment to conducting quantitative assessments to weigh the benefit–risk trade-offs. In the process of disease development, the patient's psychological factors, age, gender, etc. can also affect pain, but the current research has no way to evaluate or effectively eliminate the influence of these confounding factors.

This study focuses on how the use of anti-inflammatory drugs during the acute phase of pain affects the incidence of chronic pain and has several strengths. (1) This is the most recent review of the effects of acute-phase anti-inflammatory drug therapy on the incidence of chronic pain; (2) we conducted a comprehensive search of eligible RCTs in all languages, with no specified date, age, sex, or language restrictions, and also searched citations of relevant trial reports and reviews for potentially eligible studies; (3) all factors that may affect the results were considered in the analysis of the data. Subgroup analyses were designed according to the characteristics of the available studies, including pain type, follow-up time, drug, dose, and duration and (4) this review follows the PRISMA statement, and the procedures throughout the review process are rigorous and reproducible.

### Interpreting the findings

For neuropathic pain, analgesic antidepressants and antiepileptic drugs are first-line medications based on many placebo-controlled trials that are of moderate and high quality (Finnerup et al. 2015; Derry et al. 2013, 2014). However, there is still no consensus on the effectiveness of the prevention of chronic neuropathic pain. The current data show that the combination of anti-inflammatory drugs (methylprednisolone and dexamethasone) in the acute phase of neuropathic pain may provide greater benefits to patients. But most steroids are combined with a local anesthetic, making the results indirect. Specifically designed single-drug intervention RCTs with long follow-up are needed. Effects of inflammatory response and other factors can last for a period of time (days or weeks), and studies of a single-shot drug intervention may be difficult to find valuable results.

## Conclusion

The quality of evidence was low to moderate. Steroids or NSAIDs have analgesic effects in the acute phase but do not reduce the incidence of chronic nociceptive pain. For neuropathic pain, steroids use in the acute phase significantly reduced the incidence of chronic pain at 3–6 months (by 53%). Only methylprednisolone and dexamethasone had an observed benefit, with some adverse effects. However, the evidence is indirect and needs to be interpreted with caution.

### Supplementary Information

Below is the link to the electronic supplementary material.

Supplementary file1 (DOC 12502 KB)

## Data Availability

The analytic dataset is available on request by contacting the corresponding author.
