# Supplementary material for: Effect of use of NSAIDs or steroids during the acute phase of pain on the incidence of chronic pain: a systematic review and meta-analysis of randomised trials
Source: Inflammopharmacology. 2023 Dec 28;32(2):1039–58. doi: 10.1007/s10787-023-01405-8 (PMC11006744; doi:10.1007/s10787-023-01405-8)
Supplement: Supplementary file 1 — Supplementary file1 (DOC 12502 KB) [file 10787_2023_1405_MOESM1_ESM.doc]

**Article title:** Effect of use of NSAIDs or steroids during the acute phase of pain on the incidence of chronic pain: a systematic review and meta-analysis of randomised trials

**Journal name:** [Inflammopharmacology](https://www.springer.com/journal/12630/)

**Author names:** Luyao Huo, Gang Liu, Bowen Deng, Lin Xu, Yanjun Mo, Shengyuan Jiang, Jingwei Tao, Huizhong Bai, Li Wang, Xiaoxiao Yang, Jizhou Yang, Xiaohong Mu

**Corresponding author:** Dr. Mu Xiaohong, Department of Orthopedics, Dongzhimen Hospital, Beijing University of Chinese Medicine, Beijing, China.

E-mail: [muxiaohong2006@126.com](mailto:muxiaohong2006@126.com)


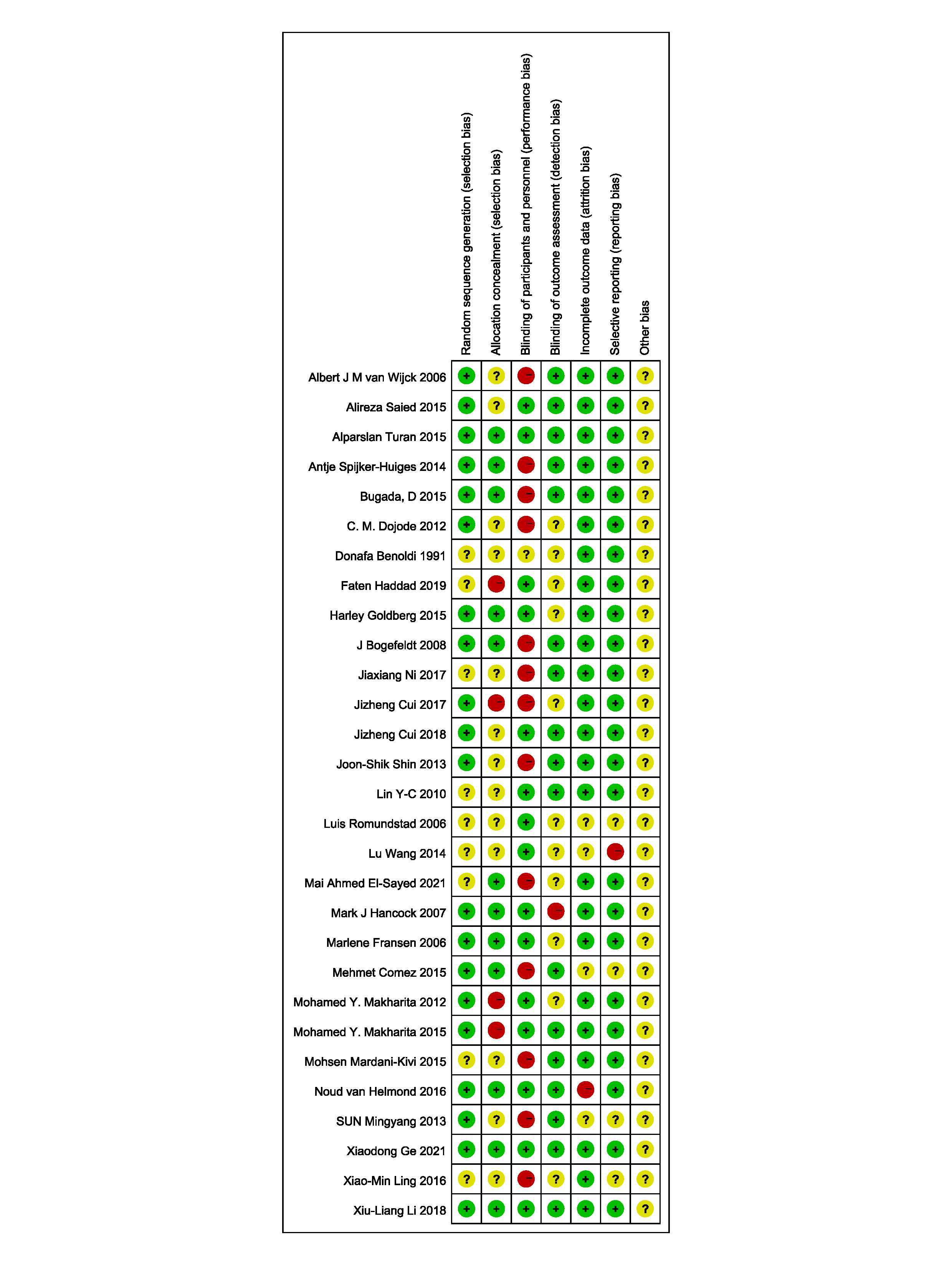


SFig. 1. Risk of bias assessment details

Note: Green: low bias risk; Red: high bias risk; Yellow: unclear judgment.


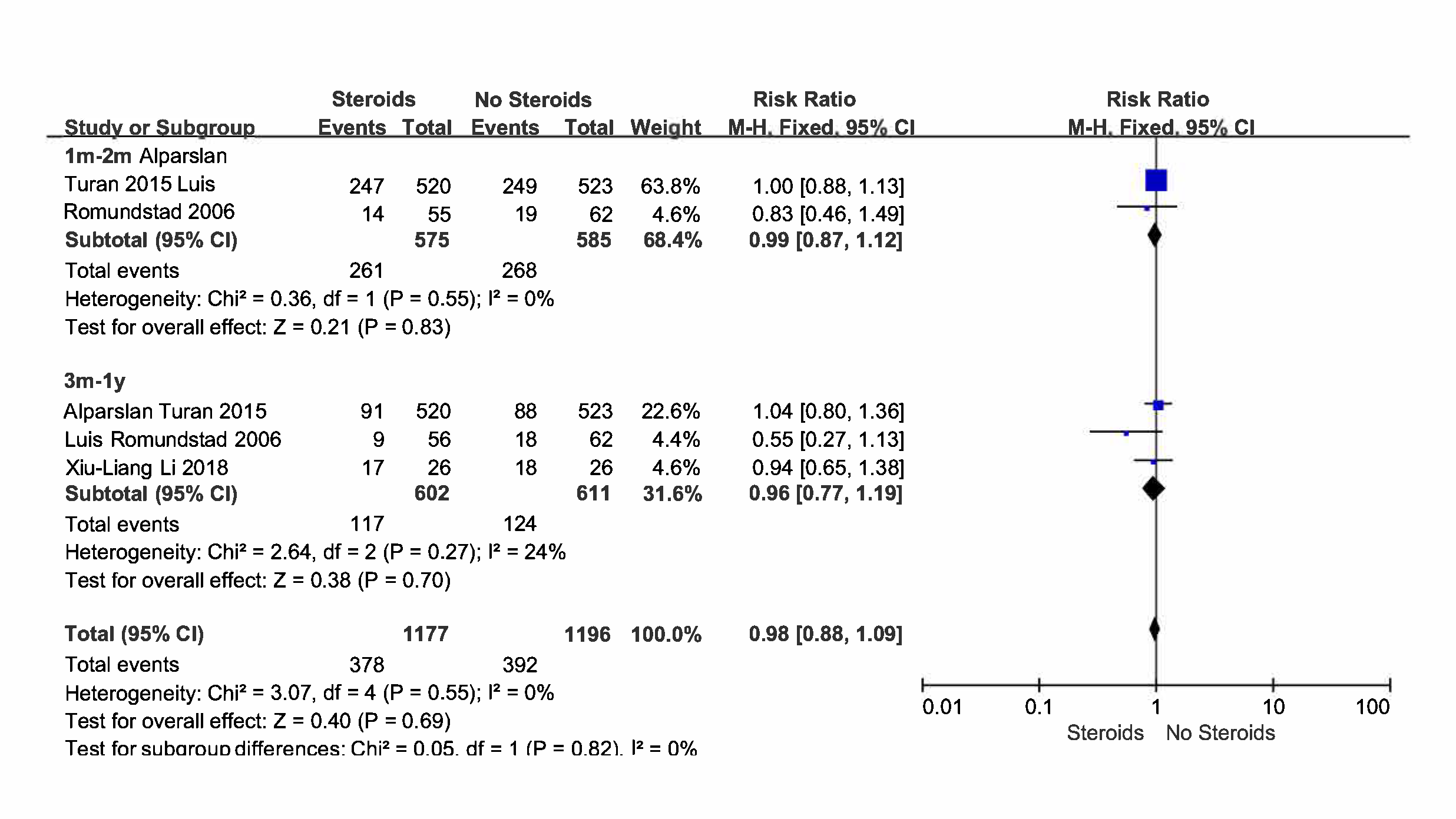


SFig. 2 Steroids and incidence of chronic postoperative pain


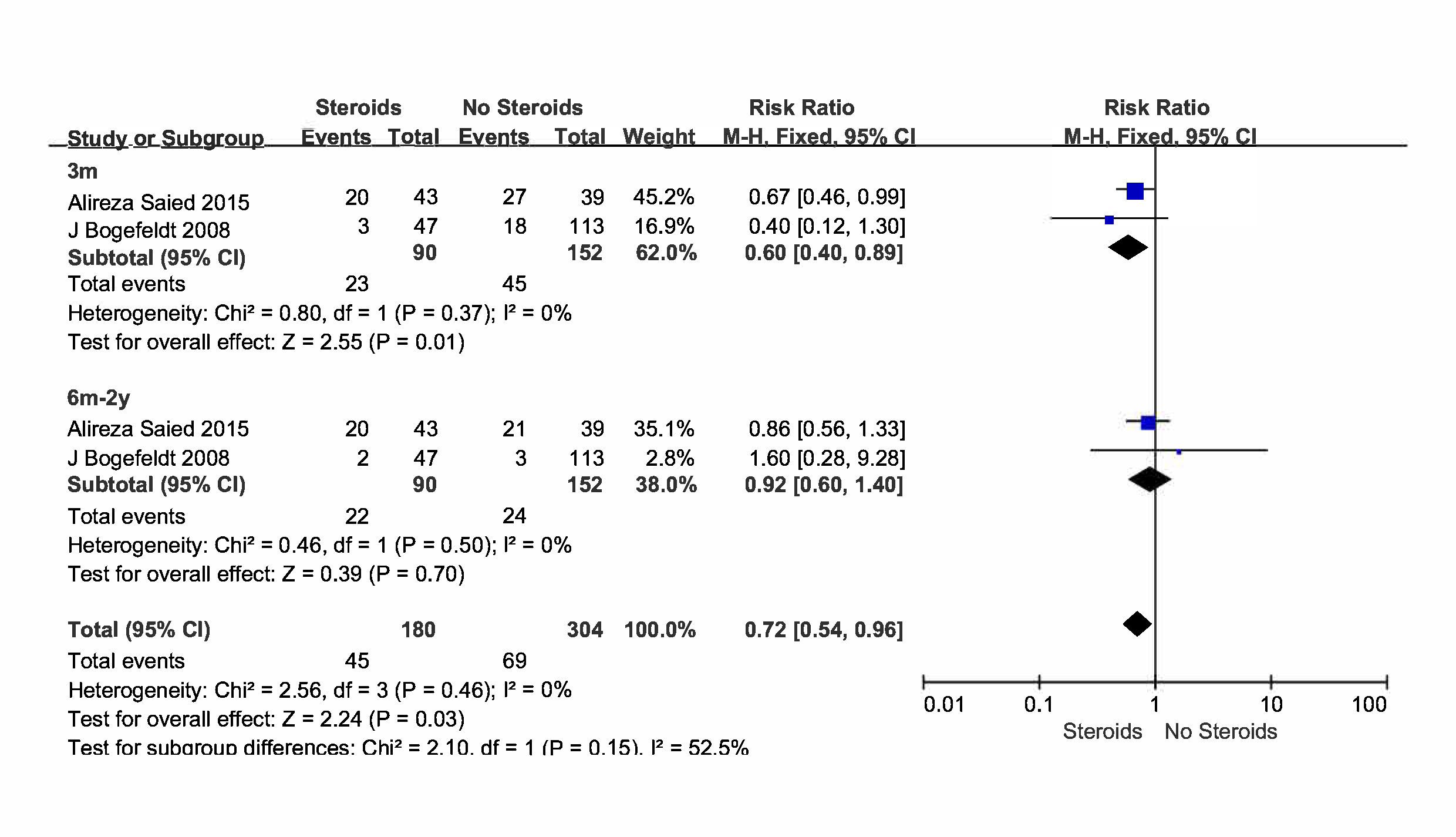


SFig. 3 Steroids and incidence of nonpostoperative chronic pain


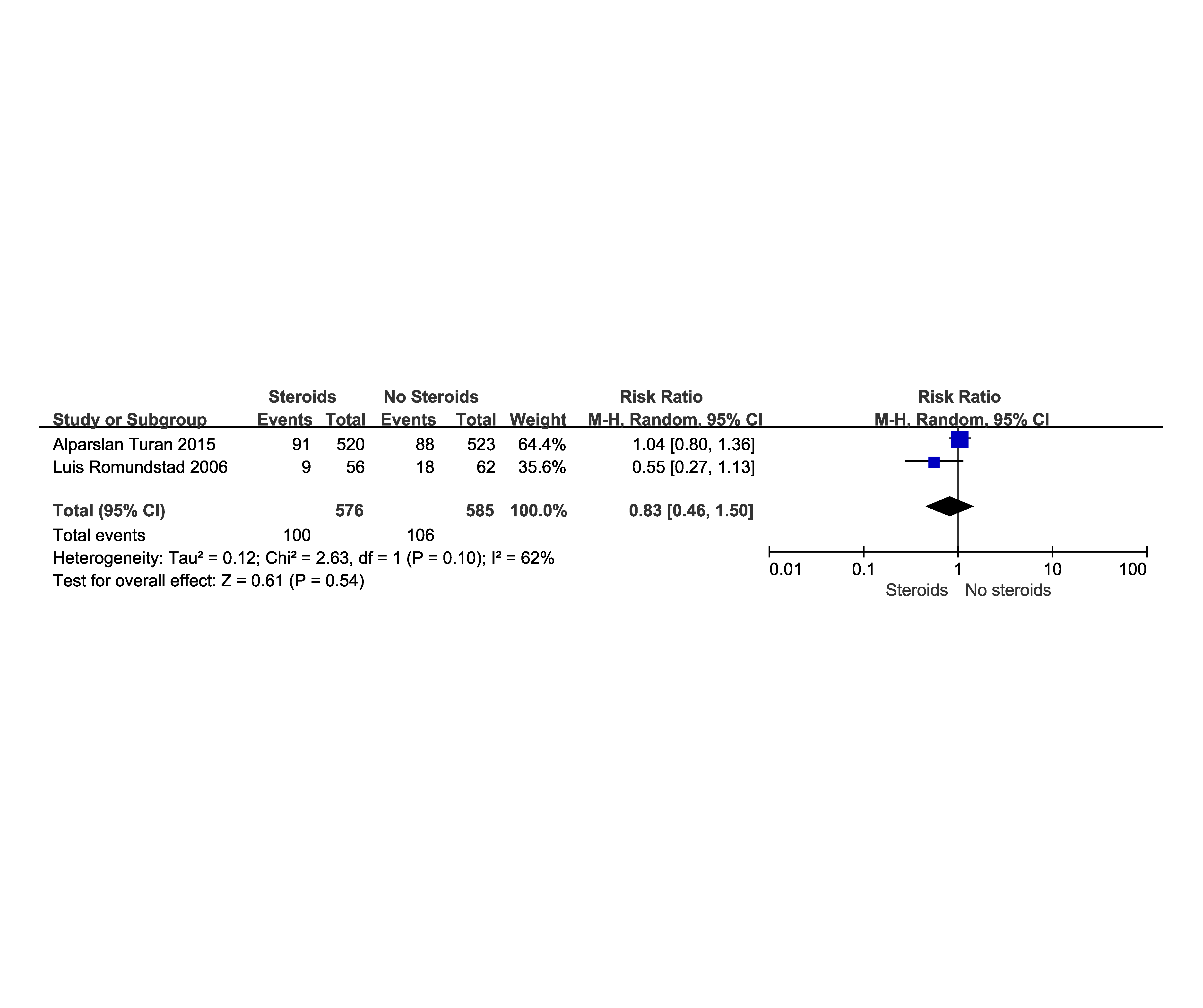


SFig. 4 Medrol and incidence of chronic nociceptive pain


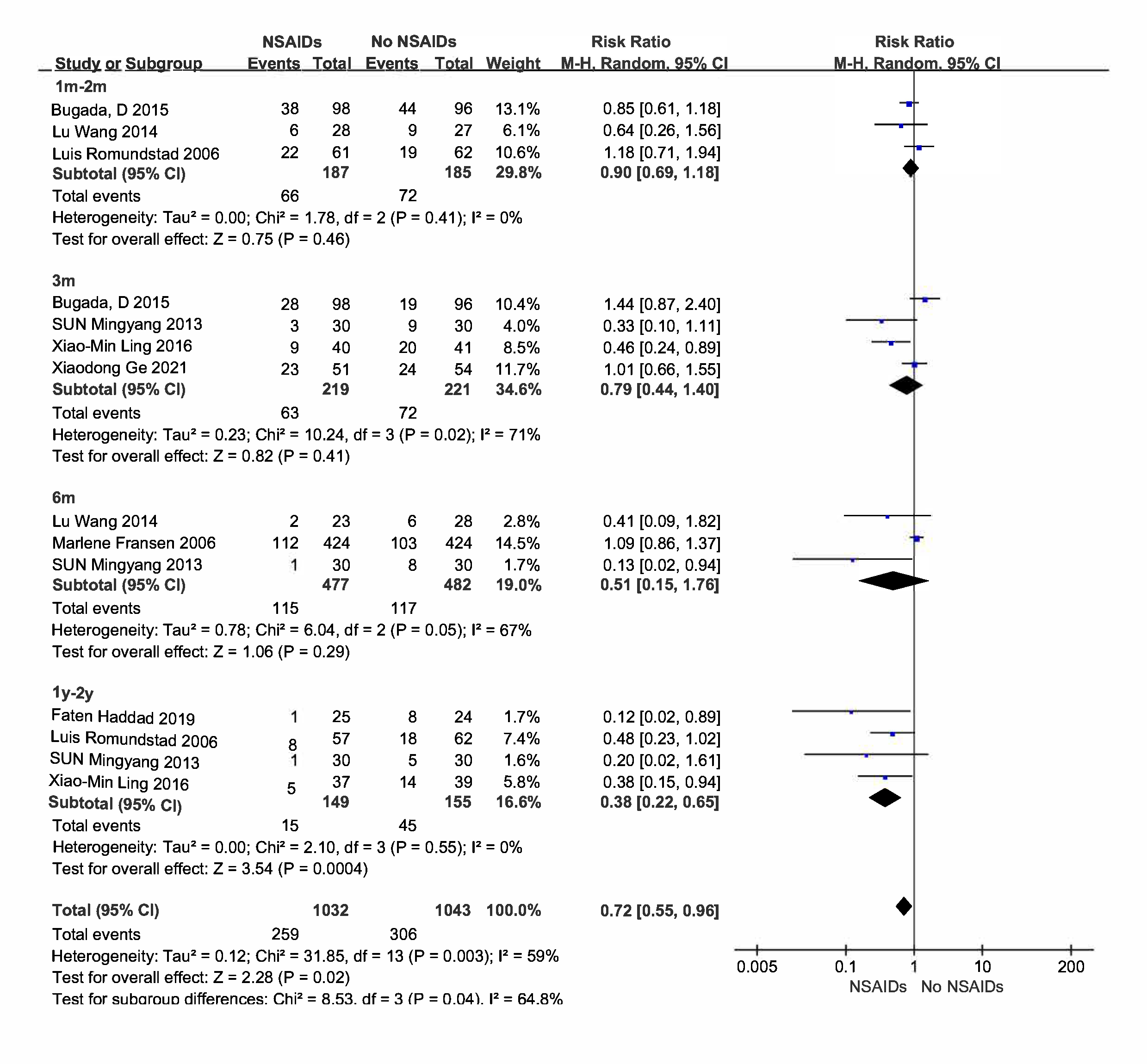


SFig. 5 NSAIDs and incidence of chronic postoperative pain


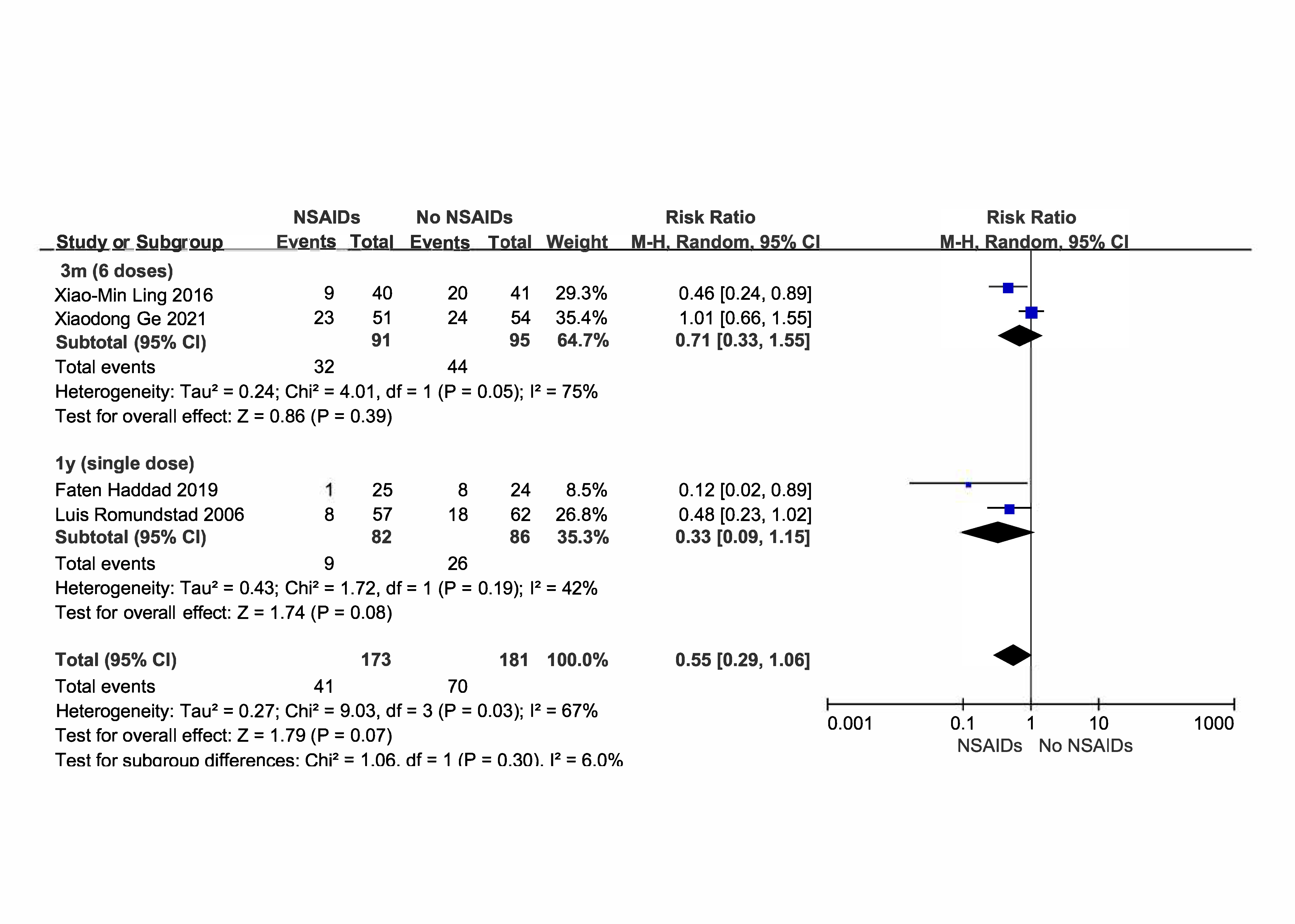


SFig. 6 Parecoxi and incidence of chronic nociceptive pain


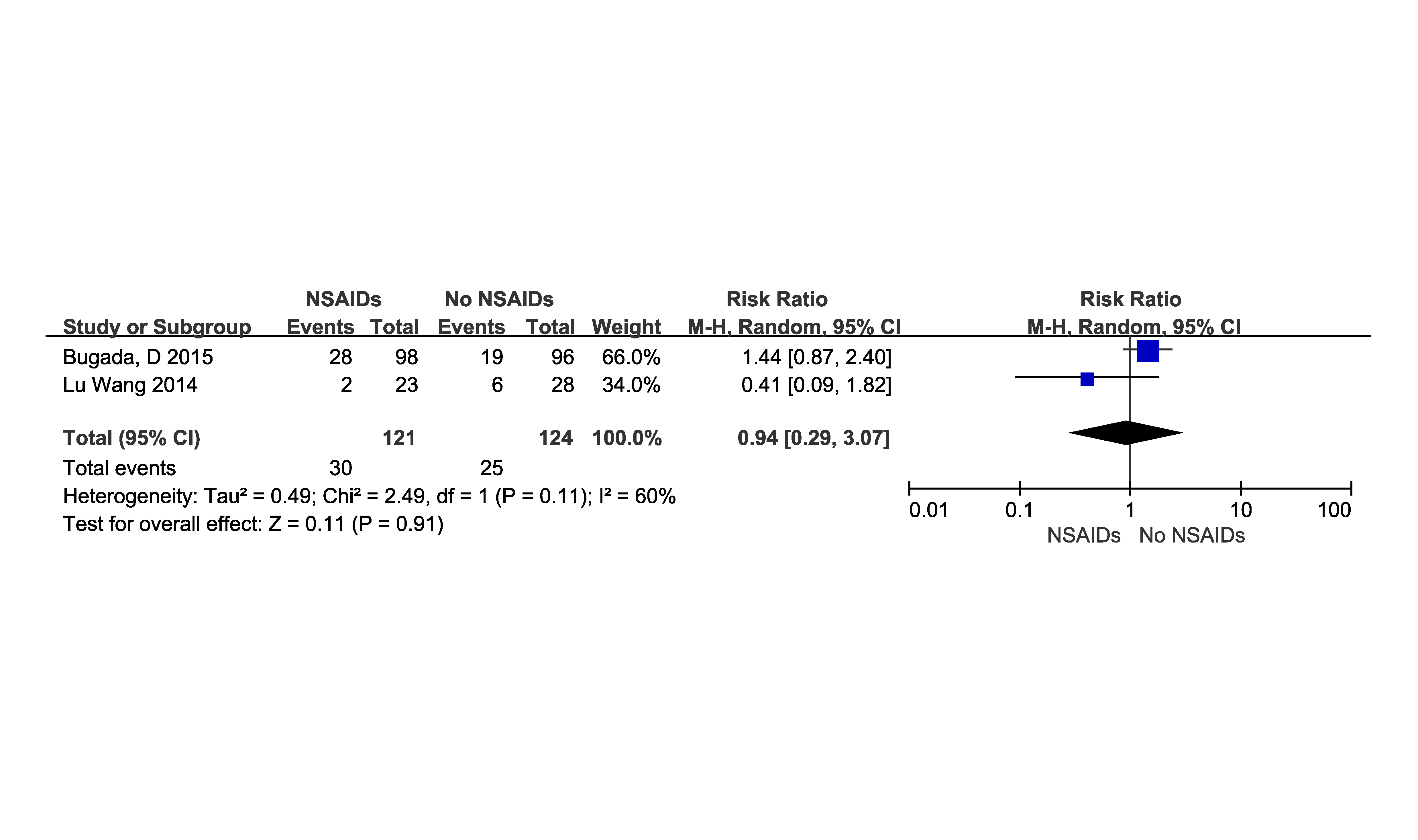


SFig. 7 Ketorolac and incidence of chronic nociceptive pain


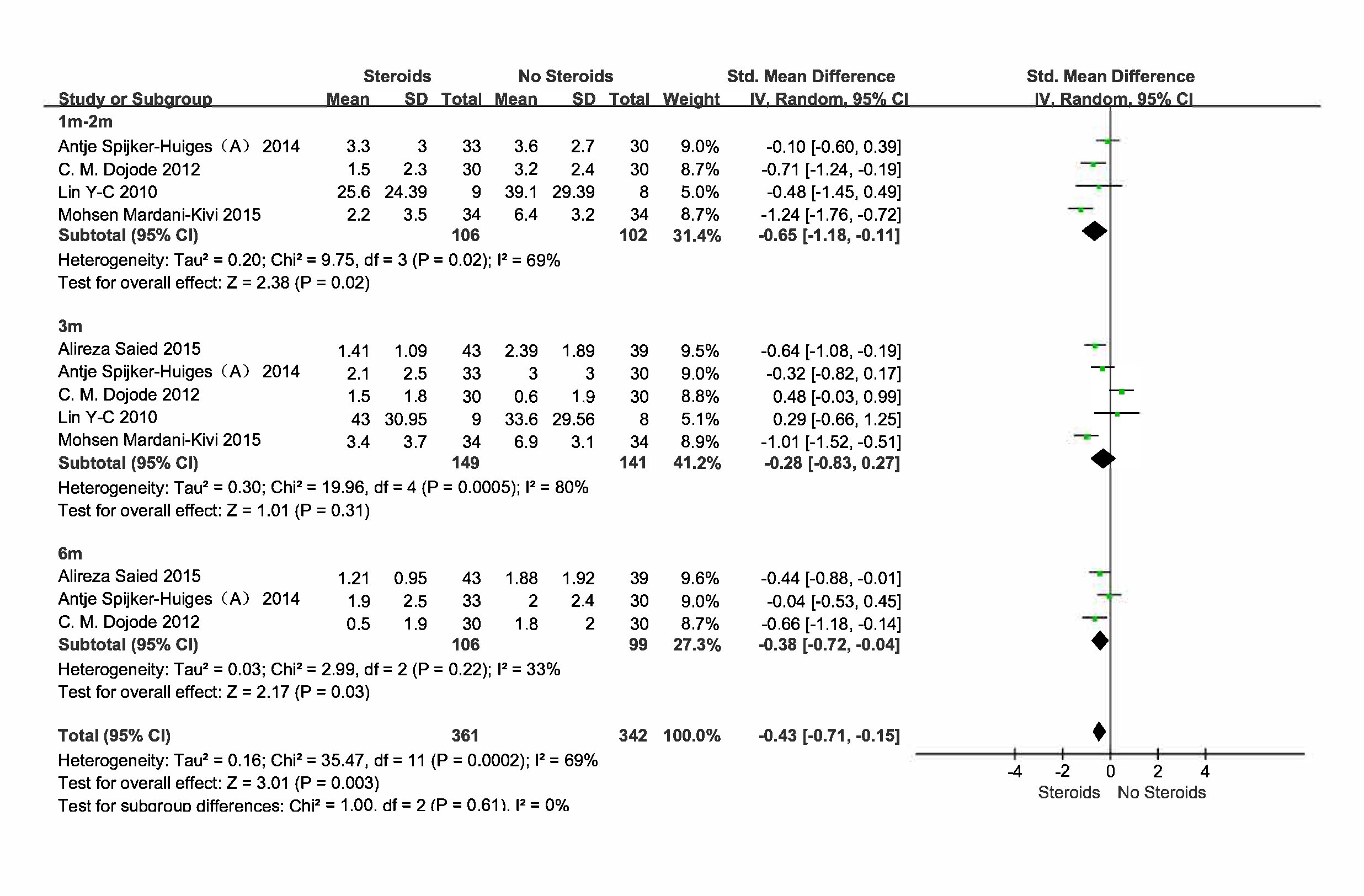


SFig. 8 Steroids and pain intensity of non-postoperative


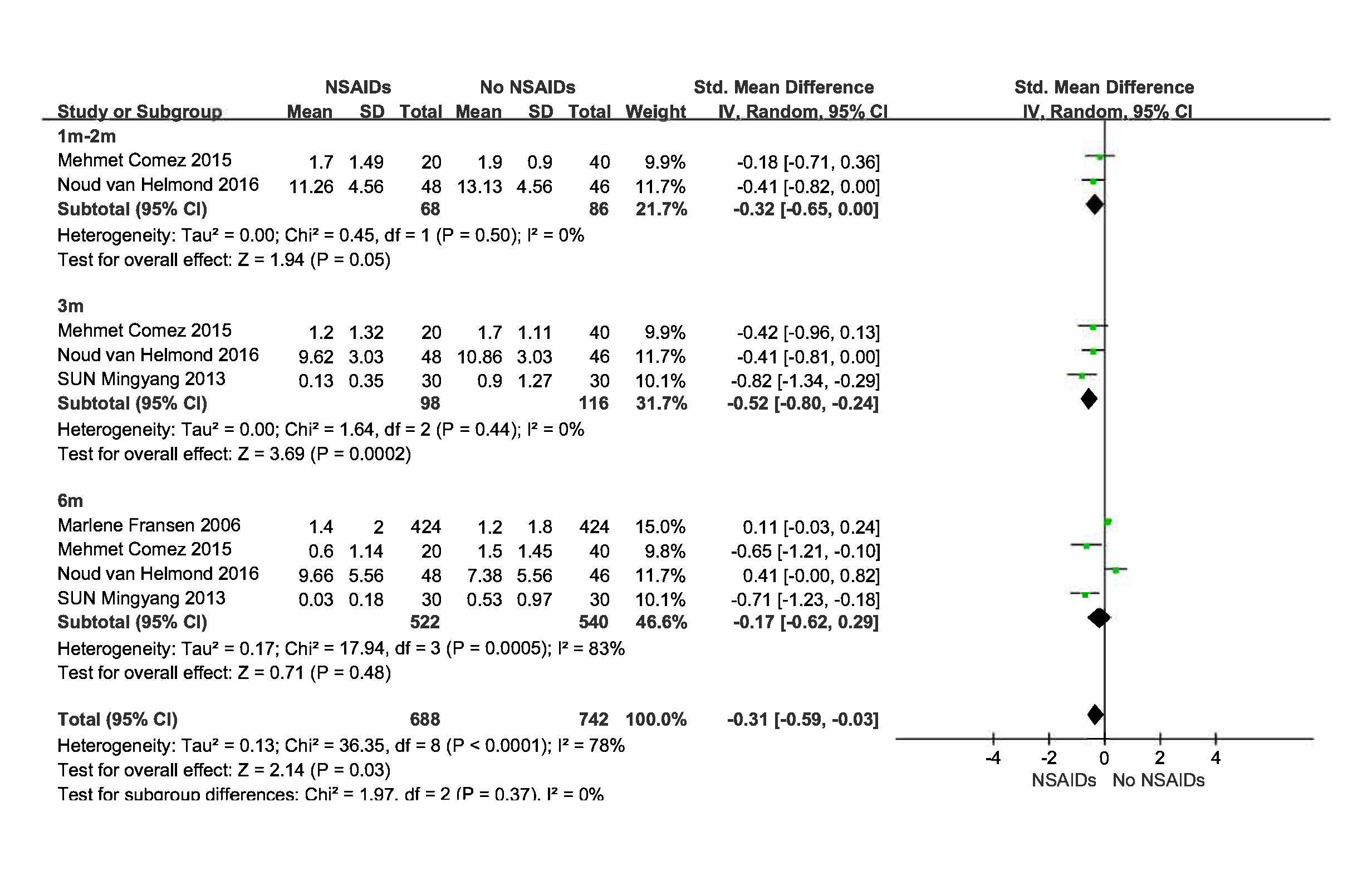


SFig. 9 NSAIDs and pain intensity of postoperative


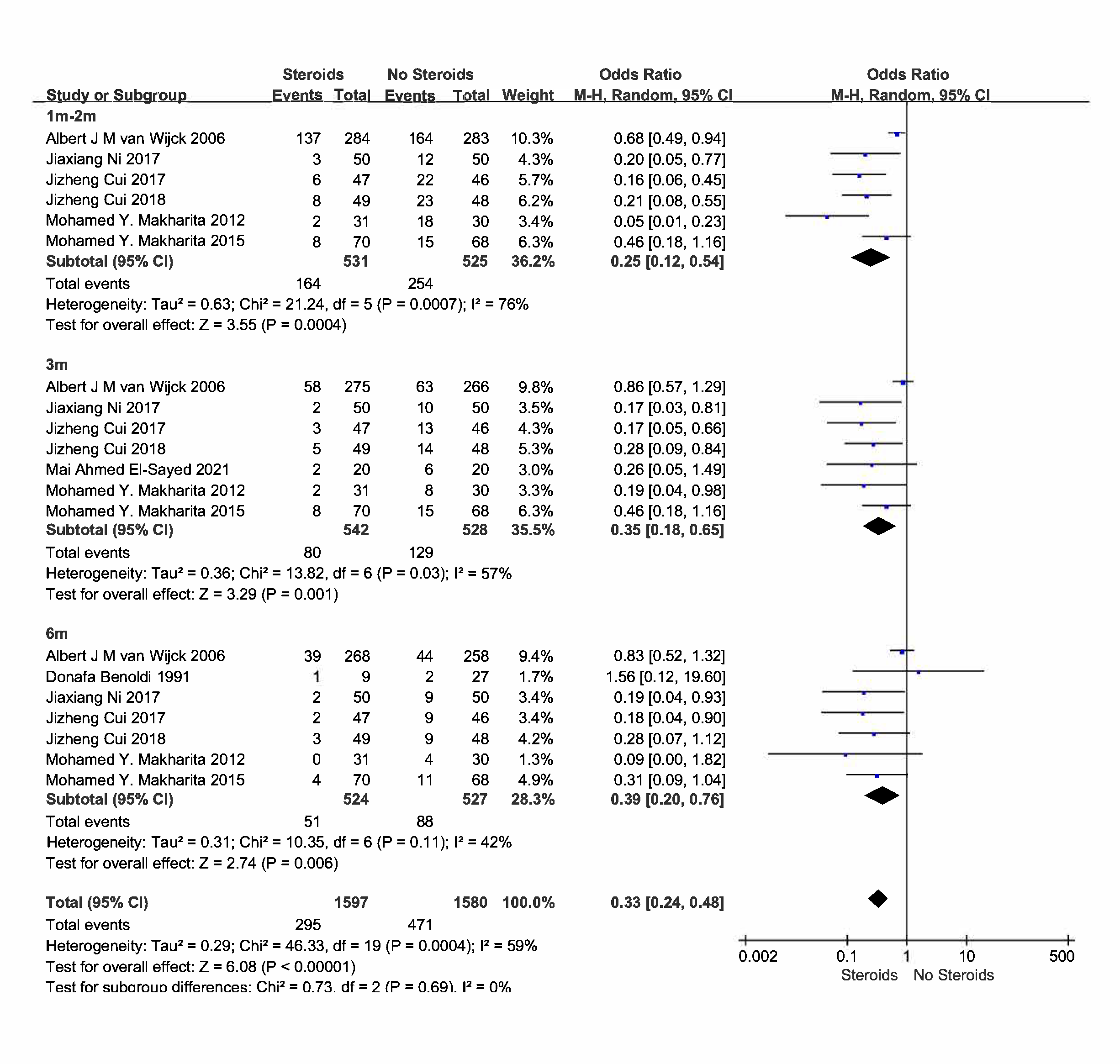


SFig. 10 Steroids and incidence of acute postherpetic pain


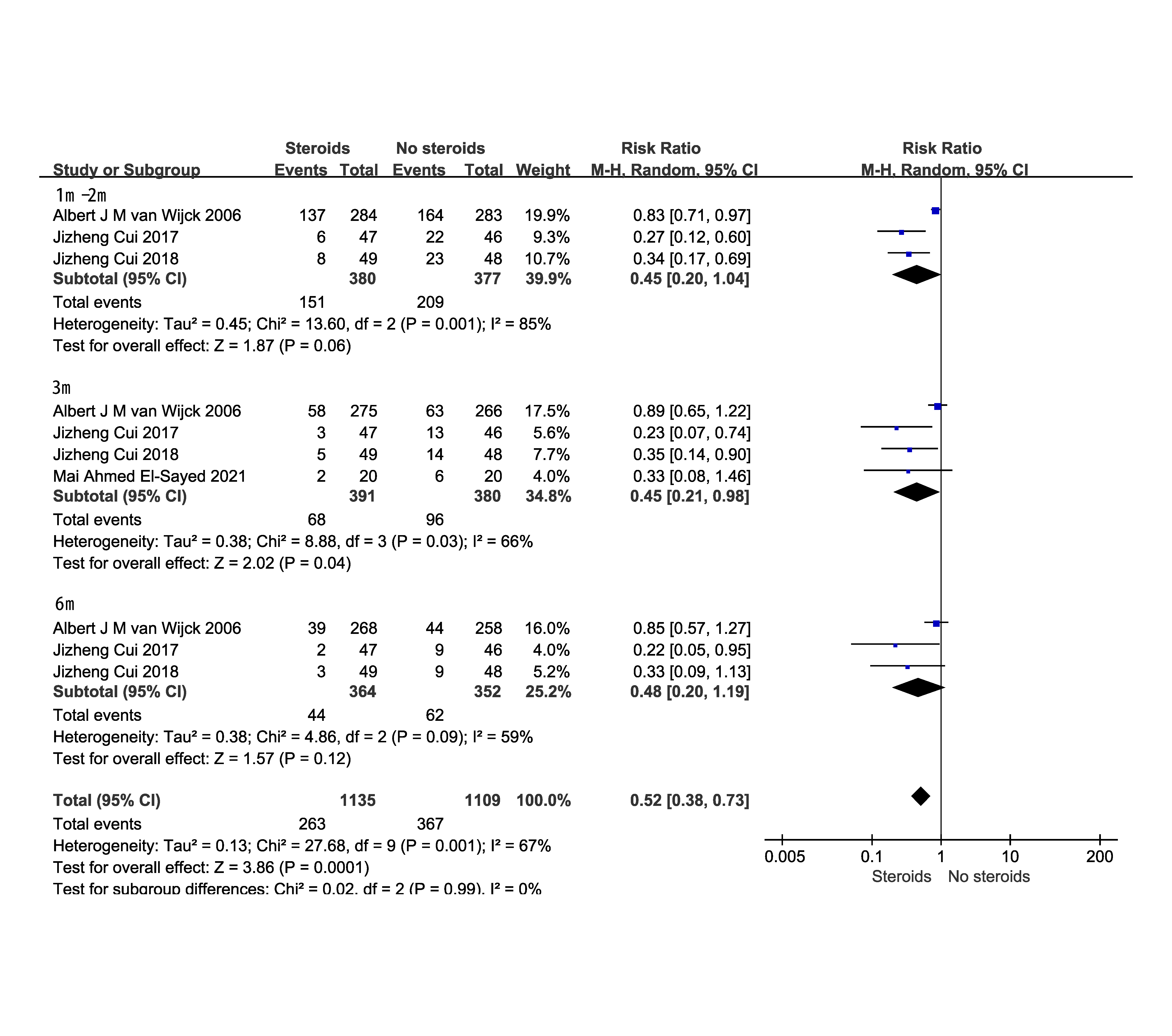


SFig. 11 Medrol and incidence of chronic neuropathic pain


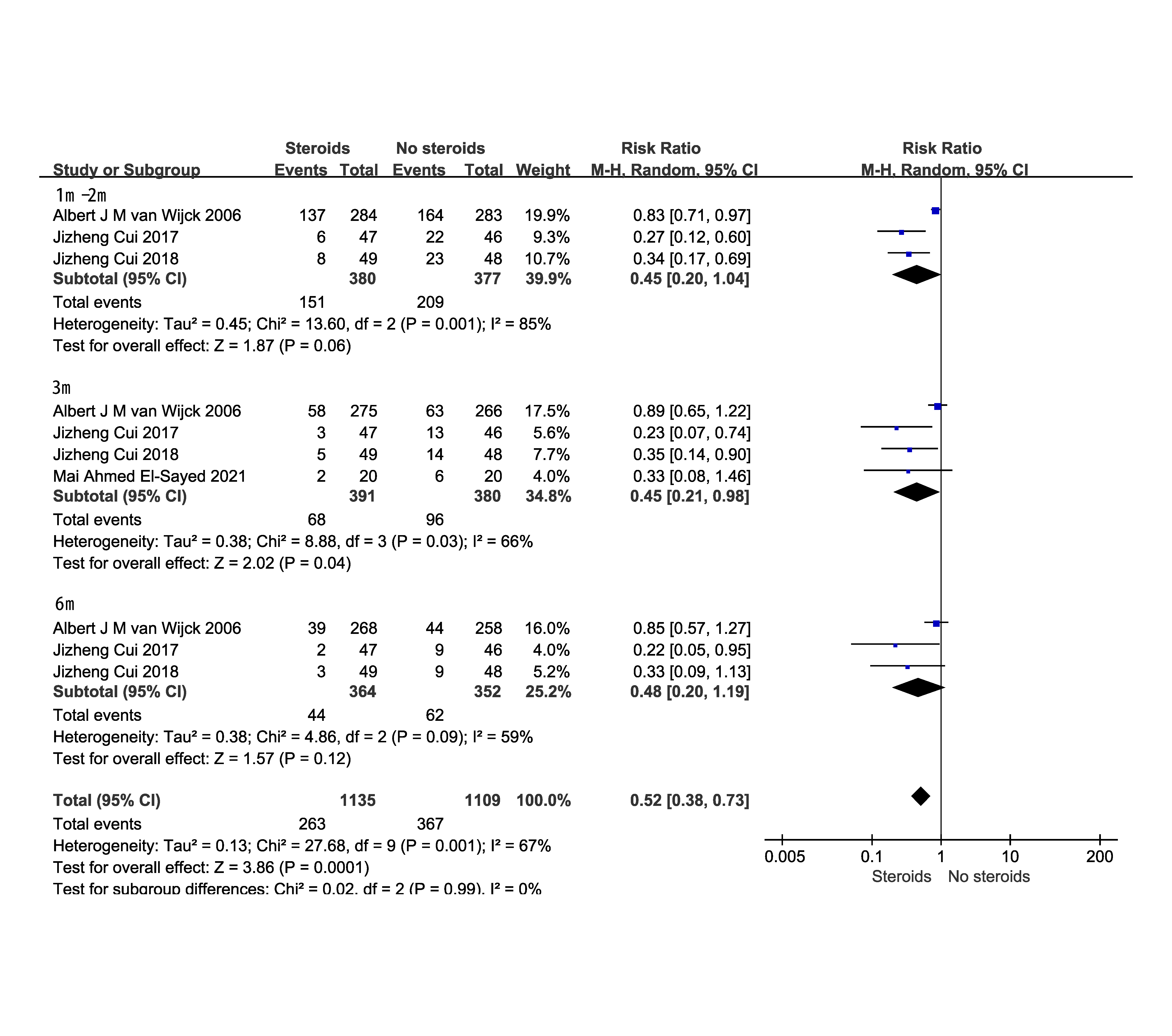


SFig. 12 Dex and incidence of chronic neuropathic pain


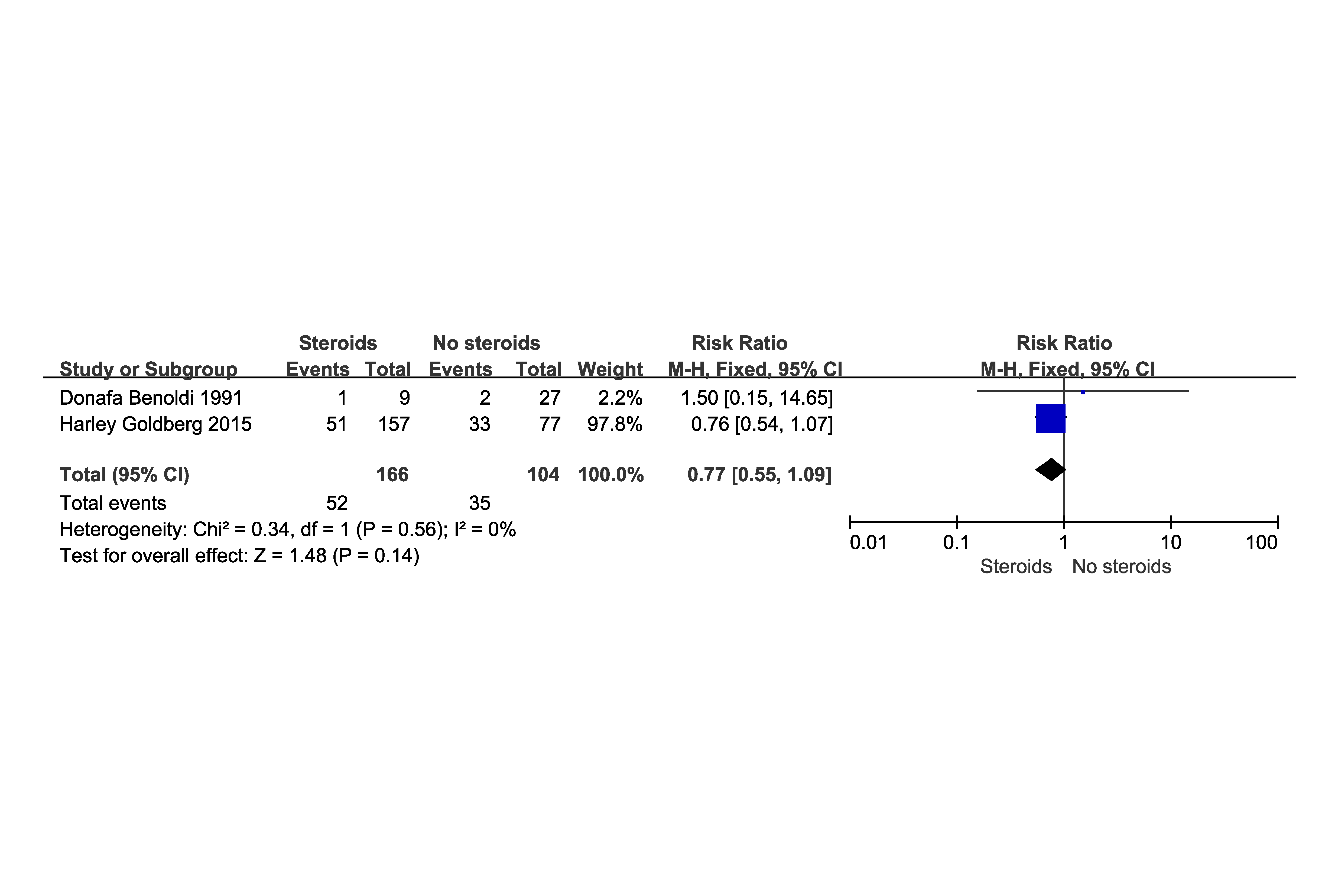


SFig. 13 PDN and incidence of chronic neuropathic pain


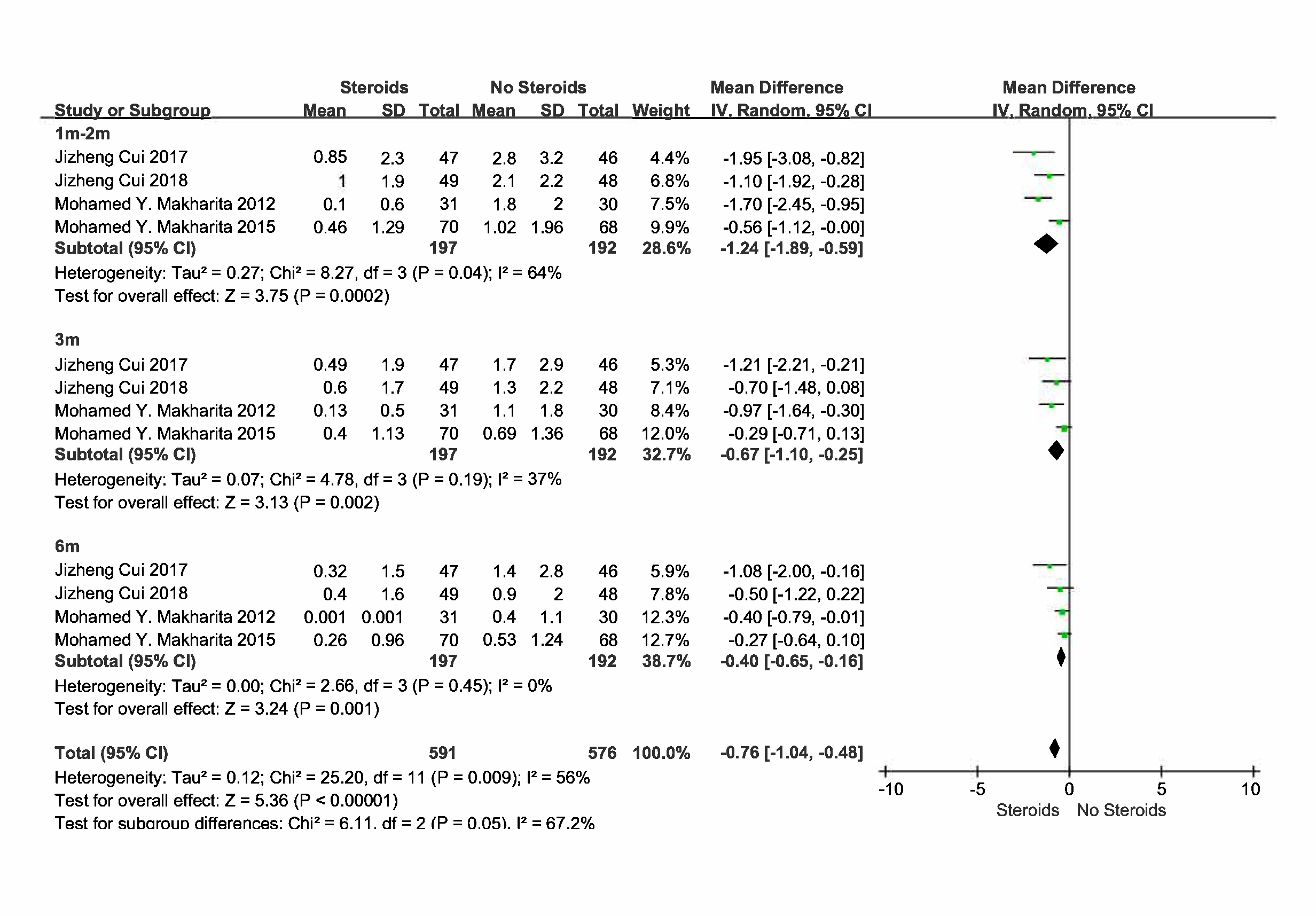


SFig. 14 Steroids and pain intensity in acute herpes zoster


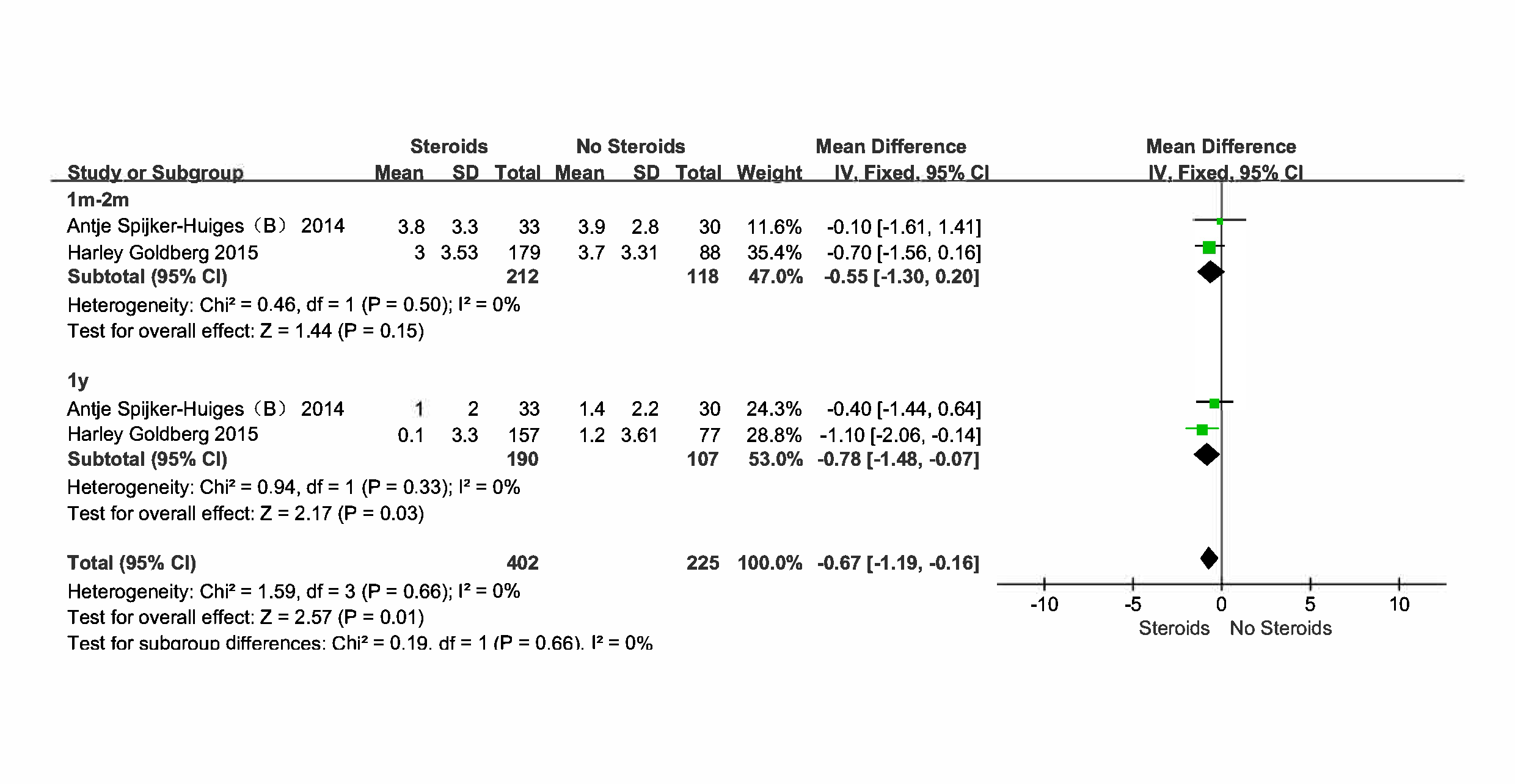


SFig. 15 Steroids and pain intensity in acute radiculopathy


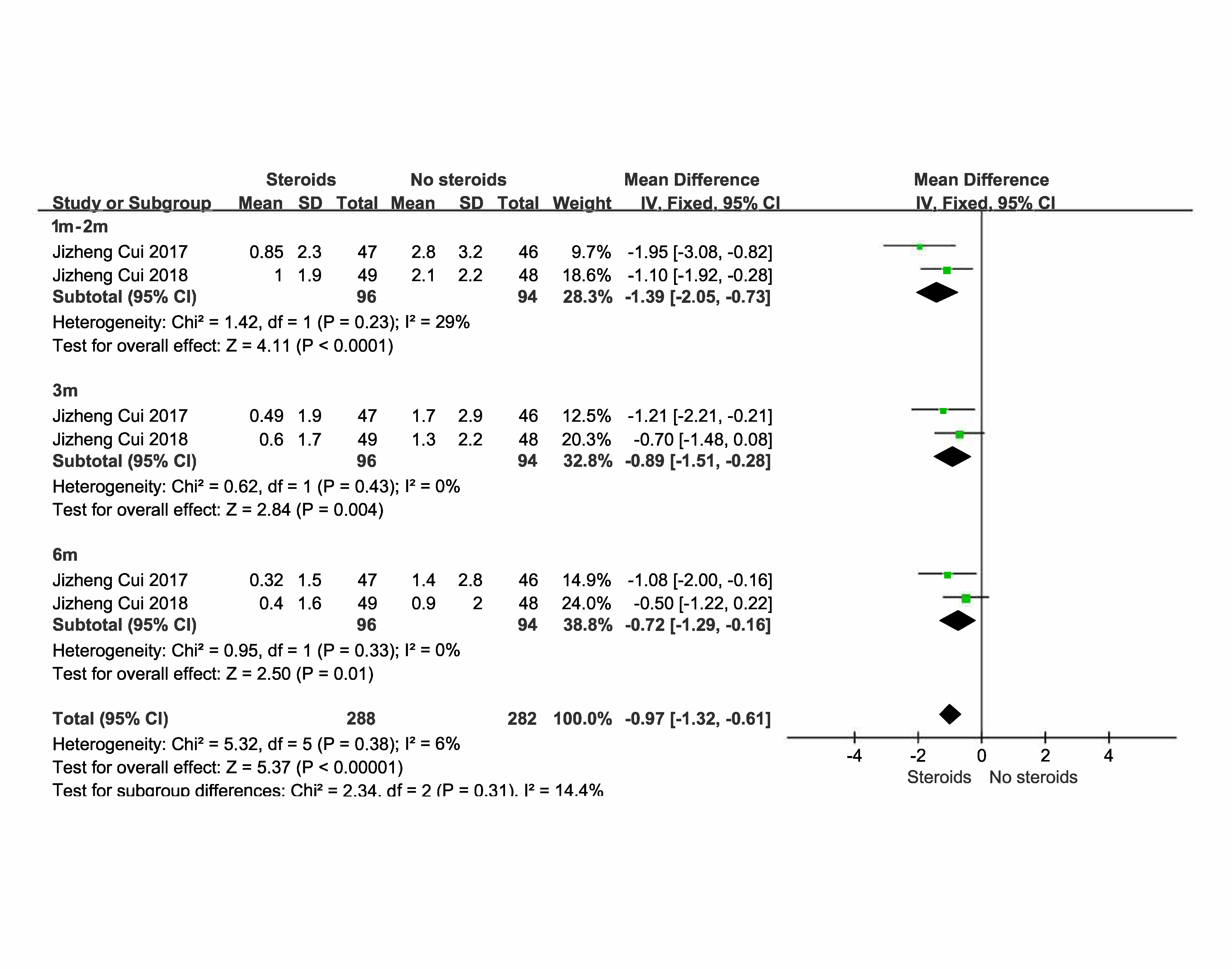


SFig. 16 Medrol and intensity of chronic neuropathic pain


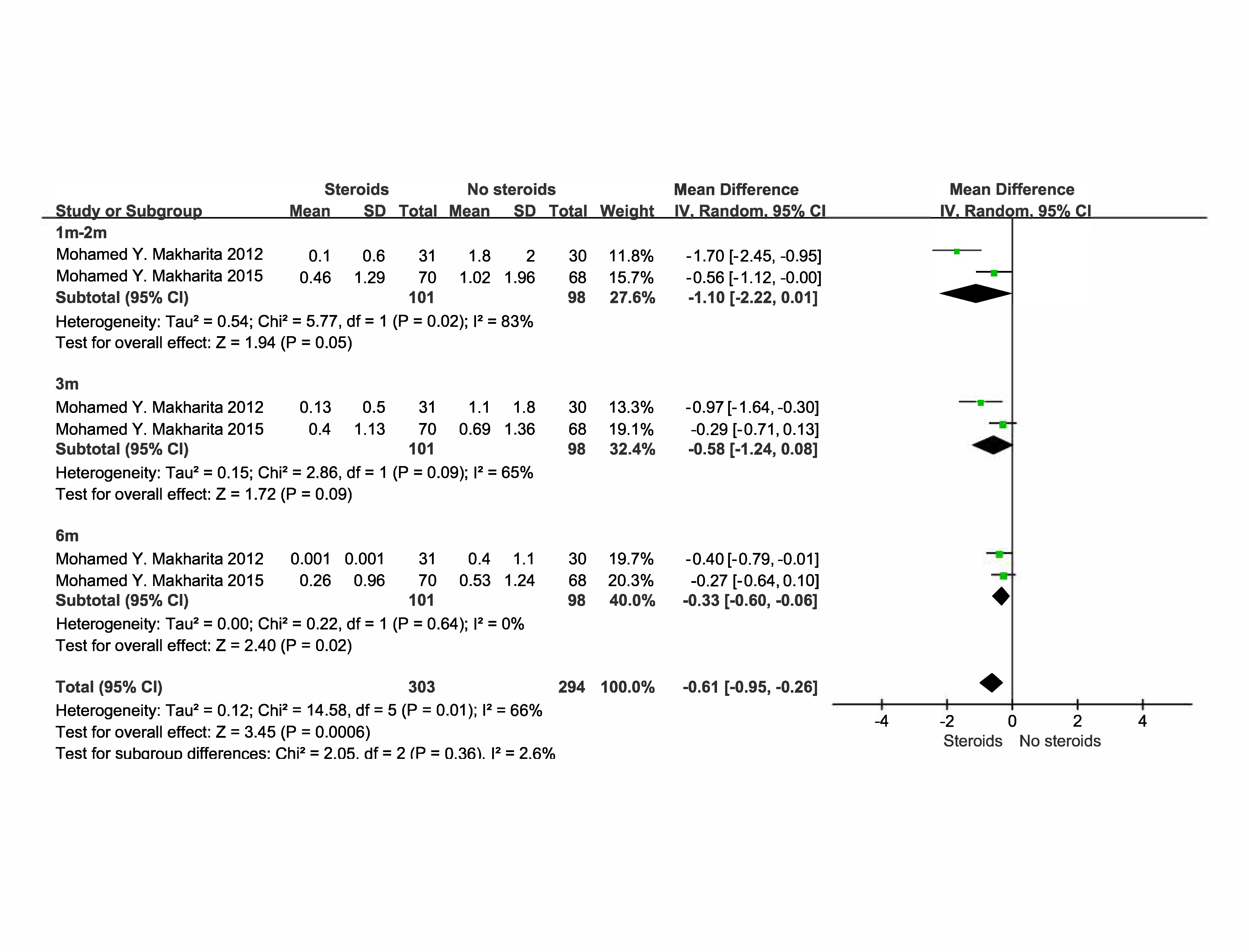


SFig. 17 Dex and intensity of chronic neuropathic pain

**Supplementary Table 1. Search terms for Cochrane Library**

Search for Cochrane Library

ID Search

#1 Acute Pains

#2 (Pain, Acute):ti,ab,kw OR (Pains, Acute):ti,ab,kw

#3 #1 or #2

#4 NSAID

#5 (Nonsteroidal Anti-Inflammatory Agent):ti,ab,kw OR (Agent, Nonsteroidal Anti-Inflammatory):ti,ab,kw OR (Anti-Inflammatory Agent, Nonsteroidal):ti,ab,kw OR (Nonsteroidal Anti Inflammatory Agent):ti,ab,kw OR (NSAIDs):ti,ab,kw RO (Antiinflammatory Agents, Non Steroidal):ti,ab,kw OR (Antiinflammatory Agents, Nonsteroidal):ti,ab,kw OR (Nonsteroidal Antiinflammatory Agents):ti,ab,kw OR (Non-Steroidal Anti-Inflammatory Agents):ti,ab,kw OR (Non Steroidal Anti Inflammatory Agents):ti,ab,kw OR (INonsteroidal Anti-Inflammatory Agents):ti,ab,kw OR (Nonsteroidal Anti Inflammatory Agents):ti,ab,kw OR (Agent, Non-Steroidal Anti-Inflammatory):ti,ab,kw OR (Anti-Inflammatory Agent, Non-Steroidal):ti,ab,kw OR (Non Steroidal Anti Inflammatory Agent):ti,ab,kw OR (Anti Inflammatory Agents, Nonsteroidal):ti,ab,kw OR (Analgesics, Anti-Inflammatory):ti,ab,kw OR (Anti-Inflammatory Analgesics):ti,ab,kw OR (Aspirin-Like Agents):ti,ab,kw OR (Aspirin Like Agents):ti,ab,kw OR (Aspirin-Like Agent):ti,ab,kw OR (Agent, Aspirin-Like):ti,ab,kw OR (Aspirin Like Agent):ti,ab,kw OR (acetylsalicyl):ti,ab,kw OR (salicylic acid):ti,ab,kw OR (carbasalate):ti,ab,kw OR (calcium):ti,ab,kw OR (diflunisal):ti,ab,kw OR (aceclofenac):ti,ab,kw OR (alclofenac):ti,ab,kw OR (diclofenac):ti,ab,kw OR (indomethacin):ti,ab,kw OR (indometacin):ti,ab,kw OR (sulindac):ti,ab,kw OR (meloxicam):ti,ab,kw OR (piroxicam):ti,ab,kw OR (dexibuprofen):ti,ab,kw OR (dexketoprofen):ti,ab,kw OR (fenoprofen):ti,ab,kw OR (flurbiprofen):ti,ab,kw OR (Ibuprofen):ti,ab,kw OR (Ibuprofen):ti,ab,kw OR (naproxen):ti,ab,kw OR (tiapro):ti,ab,kw OR (nabumeton):ti,ab,kw OR (etoricoxib):ti,ab,kw OR (phenazone):ti,ab,kw OR (propyphenazone):ti,ab,kw OR (celecoxib):ti,ab,kw OR (parecoxib):ti,ab,kw OR (cyclooxygenase inhibitor):ti,ab,kw OR (metamizol):ti,ab,kw OR (phenylbutazone):ti,ab,kw OR (rofecoxib):ti,ab,kw OR (valdecoxib):ti,ab,kw OR (lumiracoxib):ti,ab,kw OR (etoricoxib):ti,ab,kw OR (parecoxib):ti,ab,kw OR (vioxx):ti,ab,kw OR (celecoxib):ti,ab,kw OR (celebrex):ti,ab,kw OR (bextra):ti,ab,kw OR (prexige):ti,ab,kw OR (arcoxia):ti,ab,kw OR (etodolac):ti,ab,kw OR (floctafenine):ti,ab,kw OR (Meclofenamic Acid):ti,ab,kw OR (meclofenamate):ti,ab,kw OR (meloxicam):ti,ab,kw OR (oxaprozin):ti,ab,kw OR (piroxicam):ti,ab,kw OR (tenoxicam):ti,ab,kw OR (tolmetin):ti,ab,kw OR (STEROIDS):ti,ab,kw OR (Adrenal Cortex Hormones):ti,ab,kw OR (steroid):ti,ab,kw OR (corticosteroid):ti,ab,kw OR (glucocorticoid):ti,ab,kw OR (beclomethasone):ti,ab,kw OR (betamethasone):ti,ab,kw OR (budesonide):ti,ab,kw OR (cortisone):ti,ab,kw OR (dexamethasone):ti,ab,kw OR (flunisolide):ti,ab,kw OR (fluticasone):ti,ab,kw OR (fludrocortisone):ti,ab,kw OR (hydrocortisone):ti,ab,kw OR (cortisol):ti,ab,kw OR (methylprednisolone):ti,ab,kw OR (mometasone):ti,ab,kw OR (prednisolone):ti,ab,kw OR (prednisone):ti,ab,kw OR (triamcinolone):ti,ab,kw

#6 #4 or #5

#7 (randomized controlled trial):pt OR( nonrandomized):ti,ab,kw OR (placebo):ti,ab,kw

#8 #3 and #6 and #7
